# An open-label pilot study of psilocybin-assisted therapy for binge eating disorder

**DOI:** 10.1186/s40337-025-01508-3

**Published:** 2026-01-03

**Authors:** Jesse Dallery, Jennifer L. Miller, Jeff Boissoneault, Lauren Harvey, Lindsey Ives, Alexandra Knerr, Shelby Blaes, Morgan N. Ransom, Melissa Munson, James P. Gilligan, Michael H. Silverman, Peter R. Guzzo, Beverlee Loeser

**Affiliations:** 1 Department of Psychology, University of Florida, 945 Center Drive, Gainesville, FL 32611 USA; 2 Pediatric Endocrinology, Department of Pediatrics, College of Medicine, University of Florida, Gainesville, FL USA; 3 Department of Clinical and Health Psychology, University of Florida, Gainesville, FL USA; 4 Find Your Freedom Therapy, Gainesville, FL USA; 5 Department of Psychiatry, University of Florida , Gainesville, FL USA; 6 Tryp Therapeutics, Camberwell, Australia; 7 BioStrategics Consulting Ltd, Marblehead, MA USA; 8 Present Address: Department of Psychiatry, University of Buffalo, Getzville, USA; 9 Present Address: Department of Anesthesiology, University of Minnesota, Minneapolis, USA; 10 Present Address: Department of Psychology, Florida State University, Panama City, USA; 11 Present Address: Behavioral Pharmacology Research Unit, Johns Hopkins School of Medicine, Baltimore, USA

**Keywords:** Psilocybin, Binge-eating disorder, Acceptance and commitment therapy, Neuroimaging

## Abstract

Binge Eating Disorder (BED) is the most prevalent eating disorder and is associated with psychiatric comorbidities, health impairments, and decreased quality of life. Emerging evidence suggests that psilocybin-assisted therapy may promote cognitive and emotional flexibility and disrupt maladaptive behavioral patterns, making it a promising candidate for BED treatment. This open-label pilot study evaluated the feasibility, safety, and preliminary therapeutic effects of a single 25 mg dose of psilocybin administered in the context of Acceptance and Commitment Therapy (ACT)-based psychotherapy in adults with BED (*N* = 5). Primary outcomes included safety measures, and exploratory outcomes included self-reported binge eating frequency, depression, anxiety, psychological flexibility, anthropometric indices, and neuroimaging biomarkers assessed over a 14-week follow-up. Psilocybin was well tolerated, with no serious adverse events. Reductions in self-reported binge eating frequency were observed across all participants and sustained through week 14. Improvements were also noted in depression, anxiety, and psychological inflexibility. Three participants showed reductions in body mass index and waist circumference. Given the open label design and small sample size, causality cannot be inferred. fMRI analyses generated preliminary signals of change—such as increased functional activation from pre- to post-intervention in the middle frontal gyrus, angular gyrus, and supramarginal gyrus in response to processed versus unprocessed food cues. Psilocybin-assisted therapy was feasible and well-tolerated in individuals with BED. The clinical and neurobiological observations provide directions for future adequately powered trials.

**Supplementary Information:**

The online version contains supplementary material available at 10.1186/s40337-025-01508-3.

## Abstract (plain language summary)

Binge Eating Disorder (BED) is the most common eating disorder and can have a serious impact on mental health, physical health, and overall quality of life. Many people with BED struggle with repeated patterns of eating that feel hard to change, even when they want to. New treatments are needed to help people break these cycles in safe and supportive ways. This small pilot study explored whether a single dose of psilocybin, given alongside a type of talk therapy focused on values, acceptance, and behavior change, could be safely used with adults who have BED. Five adults took part in the study and were followed for about three months. The treatment was well tolerated, and no serious safety concerns were found. All participants reported fewer binge eating episodes after the treatment, and these improvements lasted throughout the follow-up period. Many also reported feeling less depressed and anxious and more able to respond flexibly to difficult thoughts and emotions. Some participants showed small reductions in weight and waist size. Brain scans suggested possible changes in how participants responded to food-related images. Because this was a small study without a comparison group, stronger conclusions cannot yet be made. However, the findings suggest this approach may be promising and worth studying further in larger trials.

**Supplementary Information:**

The online version contains supplementary material available at 10.1186/s40337-025-01508-3.

## Introduction

Binge Eating Disorder (BED) is the most prevalent eating disorder, affecting approximately 1.9% of the population [1]. BED is significantly more common in women than in men [2]. BED is characterized by recurrent episodes of uncontrolled food intake in the absence of compensatory behaviors typical of bulimia nervosa [1, 3]. BED is commonly associated with obesity, diminished quality of life, and psychiatric comorbidities such as depression, anxiety, and impulsivity [2, 6]. The etiology of BED is complex, including genetic and environmental factors as well as neuroendocrinological and neurobiological contributions. Neurobiological studies highlight that individuals with BED have abnormalities in reward processing, inhibitory control, and emotion regulation [2].

Despite a range of available treatments, long-term remission from BED remains difficult to achieve [4]. Meta-analyses show that while cognitive behavioral therapy (CBT) and pharmacotherapy yield moderate to large acute effects, many individuals relapse or continue to exhibit clinically significant symptoms post-treatment [1, 5]. Moreover, attrition and treatment resistance are common, particularly among individuals with high psychiatric comorbidity and cognitive inflexibility [6, 7]. These existing interventions have limited impact on weight outcomes, and their long-term effectiveness remains understudied and uncertain.

Psilocybin-assisted therapy has shown promising effects across several psychiatric conditions, including major depressive disorder, substance use disorders (e.g., nicotine and alcohol), obsessive-compulsive disorder, and anorexia nervosa [8–16]. These disorders share key features with BED, such as intrusive thoughts, compulsive behaviors, emotional dysregulation, and rigid, maladaptive patterns that are often resistant to standard treatments [6, 17]. In BED, these patterns include perseverative rumination about food, anxiety around eating, and episodic loss of behavioral control. A recent pilot study supports the feasibility of psilocybin-assisted therapy in eating disorders [15]. A single 25 mg dose of psilocybin was safe and acceptable for patients with anorexia nervosa, and a subset showed a robust decrease in eating disorder psychopathology. Psilocybin-assisted psychotherapy appears well suited to target the domains of dysfunction manifest in BED [18–21].

Psilocybin’s primary mechanism of action is agonism at the 5-HT2A receptor, with downstream effects on serotonergic tone and neuroplasticity [6]. These pathways are implicated in both appetite regulation and the affective and behavioral symptoms of BED [22, 23]. Animal studies have demonstrated that psilocybin reduces sucrose preference in obese mouse models, suggesting direct effects on hedonic feeding and reward processing [24]. Psilocybin has also been associated with increased levels of brain-derived neurotrophic factor [25], potentially contributing to lasting changes in behavior. Additionally, psilocybin may have epigenetic and anti-inflammatory effects that may be relevant in BED-related pathology [17, 26–28]. Such broad-spectrum neuromodulatory effects provide a compelling rationale for exploring psilocybin in BED.

Neuroimaging studies reveal that psilocybin reduces activity in the default mode network (DMN) while increasing global connectivity between intrinsic brain networks such as the salience and executive control networks [29–31]. These changes are associated with reduced rigid thinking and emotional dysregulation - neurobehavioral targets relevant to the maintenance of binge eating [6]. In BED, where disordered eating behaviors are often associated with altered neural patterns [2], psilocybin may temporarily disrupt maladaptive connectivity, allowing for new, more adaptive cognitive and behavioral repertoires.

Psilocybin-assisted therapy can induce profound shifts in consciousness and enhance psychological flexibility [32, 33]. Psychological flexibility refers to the ability to fully contact the present moment including all negative private events (thoughts, feelings, and physiological sensations), and engaging in behaviors consistent with goals and values [34]. Psychological flexibility is a central therapeutic target in Acceptance and Commitment Therapy (ACT), which was the framework adopted for the current study [32, 35–37]. Participants also often report increased openness, insight, and reduced experiential avoidance, which are factors linked to long-term behavior change [38–40]. Emotional breakthroughs and mystical-type experiences during dosing sessions have been shown to predict sustained improvements in mood and behavior [41–43].

We conducted an open-label pilot study to investigate the feasibility, safety, and potential therapeutic effects of psilocybin-assisted therapy in individuals with BED. Primary outcomes included feasibility and safety metrics, with exploratory assessments of changes in anthropometric measures, self-reported binge eating frequency, anxiety, depression, and psychological flexibility at several time points during a 14-week follow-up. The study also incorporated functional magnetic resonance imaging (fMRI) to provide preliminary assessment of the neural correlates of treatment response.

## Method

### Participants

We enrolled participants who met the Diagnostic and Statistical Manual of Mental Disorders, Fifth Edition (DSM-5) criteria for BED and were between the ages of 18 and 64 years. (Key enrollment criteria are described here, see Supplement 1 for a complete listing.) Participants were self-referred via ClinicalTrials.gov (NCT05035927). Participants provided informed consent and study procedures were approved by the Institutional Review Board.

All participants had to be judged medically stable by the Principal Investigator (JLM) based on a screening medical evaluation, physical examination, electrocardiogram (ECG), and routine laboratory tests, including blood and urinalysis. Individuals were excluded if they exhibited significant suicide risk, including suicidal ideation as indicated on the Columbia-Suicide Severity Rating Scale (C-SSRS) within the past year, or any suicidal behavior or clinical judgment indicating risk. Psychiatric exclusions encompassed diagnoses of schizophrenia spectrum disorders, major depressive disorder with psychotic features, bipolar I or II disorder, a family history of psychosis, or moderate to severe alcohol or substance use disorder per DSM-5 criteria. Individuals testing positive on a urine drug screen or breathalyzer at screening (or Day − 1, if repeated) were also excluded. Participants currently taking medications that inhibit UGT1A9 or UGT1A10 enzymes (e.g., regorafenib, phenytoin), or aldehyde/alcohol dehydrogenase inhibitors (e.g., disulfiram), as well as those taking serotonergic agents such as SSRIs, MAOIs, or serotonin-acting supplements (e.g., 5-HTP, St. John’s wort), were excluded. For participants with intermittent or PRN use of such agents, dosing was deferred until five half-lives had elapsed. Finally, those with contraindications to fMRI, where applicable, were excluded per institutional policy.

An unplanned interim analysis was performed for the first 5 participants dosed. Results of this analysis supported the potential of psilocybin-assisted psychotherapy for the treatment of BED, with a favorable safety profile in all participants. Based on these findings, the study was closed before the originally planned enrollment of 10 participants was reached.

### Therapy and dosing session procedures

**Fig. 1:**
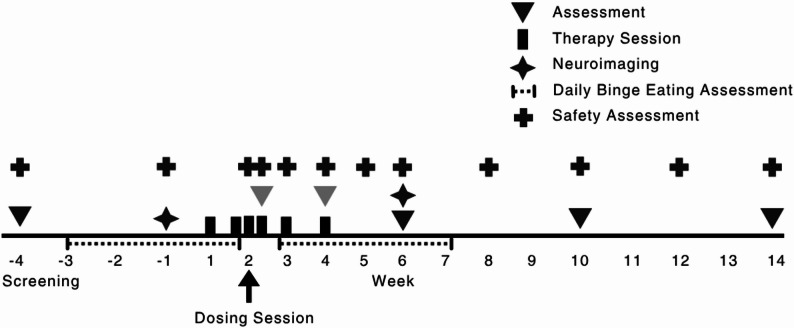
Study timeline. Note. Assessment timepoints included different batteries of measures, the partially shaded triangles indicate partial batteries. See text for further details

Figure 1 presents a timeline of study procedures and measures, and the trial protocol can be found in Supplement 1.

Two therapists conducted each therapy session, and all therapists were trained in psilocybin-assisted therapy (Fluence, Inc.) and a psilocybin-assisted therapy manual grounded in Acceptance and Commitment Therapy (ACT) for BED.

During the pre-dose preparatory therapy sessions, participants engaged in two structured meetings designed to foster therapeutic rapport, clarify expectations, provide education, and build psychological readiness for psilocybin-assisted therapy. A central theme was to adopt an attitude of openness and curiosity. The second session explored personal motivations for change and strategies for navigating challenging experiences during the dosing session. The second session was conducted in the same room as the dosing session, which was decorated and permitted adjustable ambient lighting. Participants became familiar with eyeshades, headphones for music [44], and an adjustable bed that was used during the dosing session.

Before the dosing session, staff performed a breath test for alcohol, a pregnancy test for females, and a urinalysis test to screen for recent illicit drug use. A single 25 mg oral dose of synthetic psilocybin (TRP-8802, 3-[2-(dimethylamino)ethyl]-1 H-indol-4-yl] dihydrogen phosphate developed by Usona Institute, Madison, WI, USA) was administered at approximately the same time for all participants (8:00 am). Participants were encouraged to wear eyeshades and listen to curated music to facilitate inward attention. Medical staff and rescue medications (benzodiazepines) were available on-site. At the session’s close, therapists facilitated a conversation focused on their experience and assessed for any lingering drug effects. Criteria for discharge included resolution of acute drug effects, normalized vital signs, participant self-assessment of baseline return, accompaniment by a responsible adult, and assessment and approval by the Principal Investigator (JLM).

The integration sessions focused on ACT-based strategies aligned with the participant’s emergent themes, including techniques to promote cognitive defusion, acceptance, mindfulness, and translating insights into values-based actions. The follow-up therapy session, conducted at week 4 (in-person or remote), focused on consolidating gains made during the study and supporting the participant’s continued progress after the formal intervention period.

### Measures

#### Safety

Adverse events (AEs) and serious AEs (SAEs) were tracked from consent to study completion. Vital signs, ECGs, blood chemistry and hematology, and physical exams were obtained at screening, during the dosing day (no physical exam), the day following dosing, and weeks 6, 10, and 14. During the dosing session, vital signs were obtained before dosing and at 30, 60, 90, 120, 180, 240, 300, and 360 min, and ECGs were obtained before dosing and at 60, 120, and 360 min. The Columbia-Suicide Severity Rating Scale (C-SSRS) was administered at all assessment and therapy time points except the preparation sessions, and via phone at weeks 4, 5, 8, and 12.

#### Behavioral and subjective effects

During the dosing session, both therapists completed the Monitor Rating Scale at the same intervals as the vital signs (MRS; [45], which involved ratings of the participant’s behavior and mood. Items were rated on a five-point scale (0–4). On the day following the session, participants completed three self-report measures about their experiences during the dosing session. The Mystical Experience Questionnaire (MEQ30) [46] assessed the presence and intensity of mystical-type experiences. The Emotional Breakthrough Inventory (EBI) [43] evaluated the extent to which participants experienced a psychologically meaningful release or resolution of emotion. The Challenging Experiences Questionnaire (CEQ) [47] captured the intensity and nature of difficult or distressing aspects of the experience (e.g., fear, physical distress, insanity, isolation).

#### Binge-Eating and psychological functioning

The frequency of binge eating was assessed with 2 items from the Eating Questionnaire [48]. For the 4 weeks before and the 4 weeks after dosing, participants were asked each day via smartphone: Over the past 24 h, how many times have you eaten what other people would regard as an unusually large amount of food (given the circumstances)? 2. On how many of these times did you have a sense of having lost control over your eating (at the time that you were eating)? At screening, week 10, and week 14, participants were asked the same two questions as above, but concerning the previous 28 days and with an additional question (Fairburn and Beglin, 1994): Over the past 28 days, on how many days have such episodes of overeating occurred (i.e., you have eaten an unusually large amount of food and have had a sense of loss of control at the time)? Finally, the Binge-Eating Scale (BES; [49]), a 16-item questionnaire, was used to assess the presence of binge eating behaviors at screening and weeks 6, 10, and 14. Each item has a response range from 0 to 3 points (total score < 17 indicates minimal BE problems; 18–26 indicates moderate BE problems, and a score > 27 points indicates severe BE problems).

The Hospital Anxiety Depression Scale (HADS) [50], a 14-item scale assessed depression and anxiety with two respective subscales (0–7: no or mild symptoms; 8–10: moderate symptoms; 11–14: severe symptoms; and 15–21: extreme symptoms). The Acceptance and Action Questionnaire-II (AAQ-II; Bond et al., 2011) assessed psychological flexibility. Higher scores mean greater inflexibility; scores above 25 may affect an individual’s well-being (Bond et al., 2011). Measures were administered at screening, weeks 4, 6, 10, and 14, and the HADS was also administered before and after dosing on the dosing day.

Patient improvement was also assessed relative to baseline using the Patient Global Impression – Improvement (PGI-I) and the Clinician Global Impression – Improvement (CGI-I) scales [51]. Both were a single question that asked the participant/clinician to rate the participant’s condition as compared to baseline from a 7-point scale, from [1] very much improved to [7] very much worse. Measures were administered at weeks 6, 10, and 14, and the PGI was also administered at week 4.

#### Anthropometric and biomarker measures

BMI and waist circumference were measured at screening, the dosing day, and weeks 6, 10, and 14. Blood samples were collected for metabolic biomarkers including leptin, ghrelin, insulin, and glucose before the medication dosing, and at weeks 6, 10, and 14.

### Neuroimaging

fMRI was performed once during the week of the preparation session (pre-dose) and at week 6. Neuroimage acquisition occurred after fasting from midnight (except for water). Participants were positioned head-first in the supine position in a 3T Philips Ingenia Elition X MRI scanner with a 64-channel head coil (Koninklijke Philips N.V., Amsterdam, Netherlands).

First, a blood oxygen level-dependent (BOLD) resting state functional scan (~ 9 min) was performed with the following parameters: field of view = 216 × 216, voxel-wise resolution = 2.25 × 2.25 × 2.4 mm, 54 axial slices, flip angle = 52º, multiband factor = 3, repetition time = 1.5s, echo time = 30 ms. Participants were instructed to stay as still as possible, let their thoughts wander, keep their eyes fixated on a central cross displayed on a display, and do their best to stay awake. Resting state data were not analyzed as a component of this report.

Next, participants completed a food cue reactivity task during BOLD functional imaging acquisition [52]. Sequence parameters were identical to those used in the resting state scan. During this task, participants viewed images of highly processed foods (e.g., pizza; 37.3%), minimally processed foods (e.g., apple; 29.4%), and neutrally valent pictures of household objects (e.g., light bulb; 33.3%) and were instructed to think about how much they desired each item. Each cue image was presented for 4 s with a jittered interstimulus interval consisting of a fixation cross (M_ISI_=4s, SD = 1.75s; Schulte et al., 2019). This task required approximately 14 min.

After task completion, participants were withdrawn from the MRI bore and provided with a small meal (i.e., meal replacement bar and no more than 12 ounces of water [fed state]). Once the meal was completed, they were repositioned in the MRI and the hunger VAS, resting-state scan and cue reactivity tasks were repeated. The order of the resting state scans and cue reactivity tasks were counterbalanced. Finally, a high-resolution T1-weighted structural brain image (~ 4 min) was collected with the following parameters: field of view = 225 × 288 × 288 mm, voxel-wise resolution = 1 × 0.78 × 0.78 mm, flip angle = 8º, repetition time = 6.68ms, echo time = 3ms. In total, MRI testing required approximately 1 h.

fMRI data were preprocessed and denoised using a standard pipeline within the CONN toolbox (Whitfield-Gabrieli and Nieto-Castanon, 2012). Briefly, this included realignment, slice timing correction, outlier detection using the Artifact Detection Toolbox (ART), segmentation, normalization to MNI space, and smoothing using an 8 mm kernel. Outlier volumes were those where the global signal exceeded 5 standard deviations or total displacement from the prior volume exceeded 0.9 mm. Data were denoised at the first level by regressing out the first 5 principal components from white matter and CSF; translation (x/y/z) and rotation (pitch/roll/yaw) parameters; and outlier volumes.

### Statistical analysis

Analyses focused on safety metrics and idiographic, graphed changes in binge eating frequency, psychological functioning, and anthropometric measures from baseline through follow-up. Due to the small sample size and feasibility focus, no inferential statistical tests on these outcome measures were planned.

To assess effects of time (pre- to post-treatment), satiety state (fasted vs. fed), and cue type (processed food vs. unprocessed food vs. neutral) on functional activation associated with cue presentation, whole brain GLM was conducted in SPM12 (Ashburner et al., 2014). Following convolution of the canonical hemodynamic response function with the food cue reactivity task paradigm at the single-subject level, differences in activation related to the viewing of each stimulus type were evaluated. Analyses were false discovery rate (FDR) corrected at the cluster level (pFDR < 0.05). The cluster forming threshold was *p* < .001 (uncorrected).

## Results

Eleven participants were assessed for eligibility. Six were excluded (concurrent medications, *n* = 3; psychiatric issues, *n* = 1; no documented or demonstrated diagnosis of BED, *n* = 2). Since BMI was not an inclusion/exclusion criterion, Participant 3 was included in the study despite a relatively low BMI. She denied compensatory behavior to prevent weight gain (e.g., vomiting). One participant initially failed screening due to persistent hypertension. Following initiation of propranolol treatment and after maintaining blood pressure stability for three months, the participant was rescreened, met eligibility criteria, and subsequently enrolled in the trial. Five participants, four women and one man, completed the trial. Table 1 shows demographic and key baseline characteristics.

### Safety

All AEs were judged mild and recovered except for mild sweating (P1) and an increased number of hypoglycemic events (P2). Two participants (P3 and P6) experienced a mild headache, which was attributed to psilocybin administration. No participant reported suicidal ideation or risk during the study. Peak heart rate, vital signs, and percentage elevations from pre-dose during the dosing session are shown in Table 2 [53]. No abnormal or clinically significant ECG values were obtained at any timepoint or during the dosing session. No abnormal or clinically significant laboratory analyte values were observed at any of the sampling time points.

### Behavioral and subjective effects

Table 2 reports the MRS estimates during the dosing session. All values are reported as peak effects (Griffiths et al., 2016). The results from the monitor rating scale are the average ratings across both therapists/monitors. The monitor rating scale reflects individual differences in the drug effect, from low (P2 and P3) to more substantial (P1, P5, P6) across various behaviors and emotions. Table 2 also shows the scores from the three self-report questionnaires about the psychedelic experience. One response on the CEQ for P6 was imputed based on the mean of the other responses in the subscale for that item. The scores on the CEQ were variable: three participants reported few challenging experiences (P1, P2, and P5), while P3 and P6 reported more challenging experiences. Scores for two participants (P1 and P6) on the MEQ were relatively high, and P6 was the only participant to report a “complete” mystical experience (> 60% on all four subscales, data not shown). The scores for the other three participants were relatively low. All but one participant (P3) had meaningfully high scores on the EBI.

### Binge-eating and psychological functioning

**Fig. 2:**
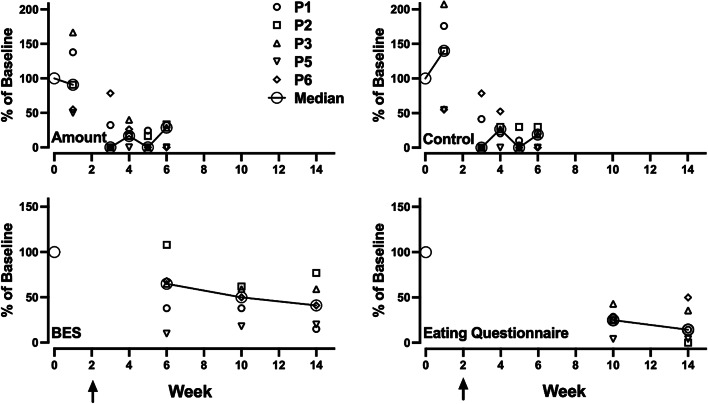
Measures of binge-eating across all timepoints. Note. Amount = Large amount of food; Control = Sense of having lost control over eating BES = Binge-Eating Scale. Arrows indicate psilocybin administration

Figure 2 shows the results of the daily assessment of binge eating during the four weeks before and four weeks after dosing. Measures are percentages relative to the average of the three weeks before any therapy (i.e., before the first preparation session). The absolute values for this period are shown in Table 1. Weekly averages were computed for each subsequent time point and compared to the three-week value. The weekly time points were the first preparation session, the first integrations session (week 3), and then weeks 4, 5, and 6. All participants reported reductions in eating a large quantity of food and loss of control overeating compared to baseline. Although some reductions occurred during the week of the preparation session for some participants, further reductions were evident post-dose for these participants.

**Fig. 3:**
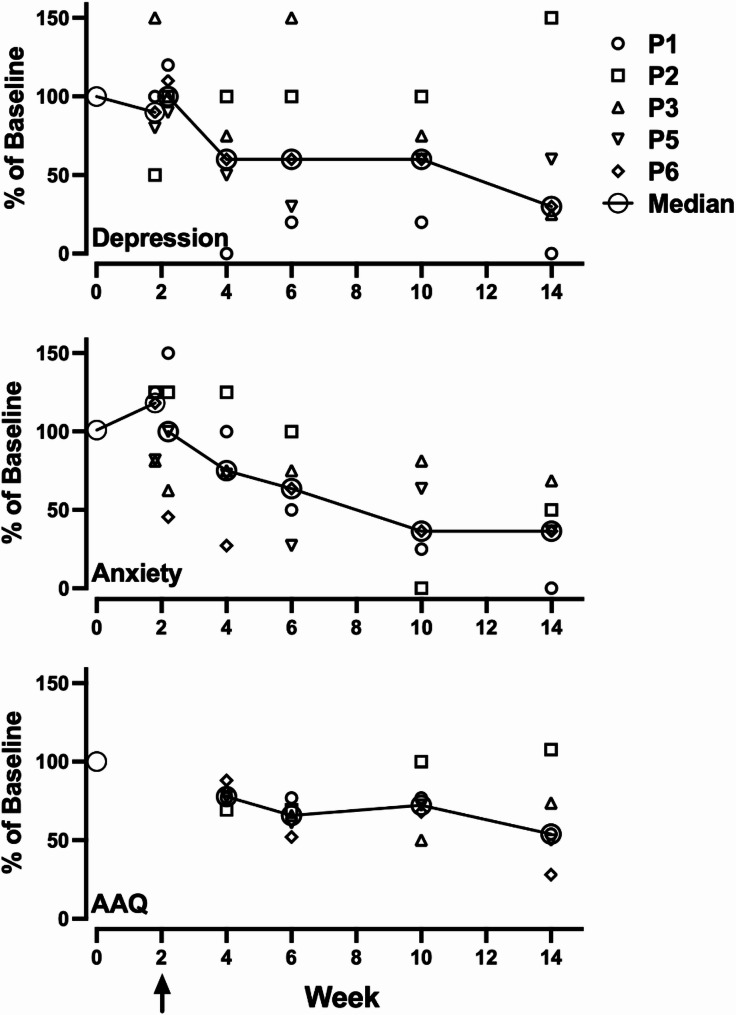
Measures of psychological functioning across all timepoints. Note: AAQ = Acceptance and Action Questionnaire. The arrow indicates psilocybin administration

Figure 3 also shows the results from the binge eating survey and the response to the third question on the eating questionnaire. There was little variability across the three questions comprising the eating questionnaire for each participant, and thus for clarity, only the responses to the third question are shown. The questionnaire measures also reflect a decrease relative to the screening values (see Table 1 for absolute values) and individual variability. For example, P1 did not show a decrease in the BES at week 6 and generally showed higher levels than the other participants at the week 10 and 14 time points. Both measures, however, reflect sustained decreases in self-reported binge eating through the 14-week follow-up.

**Fig. 4:**
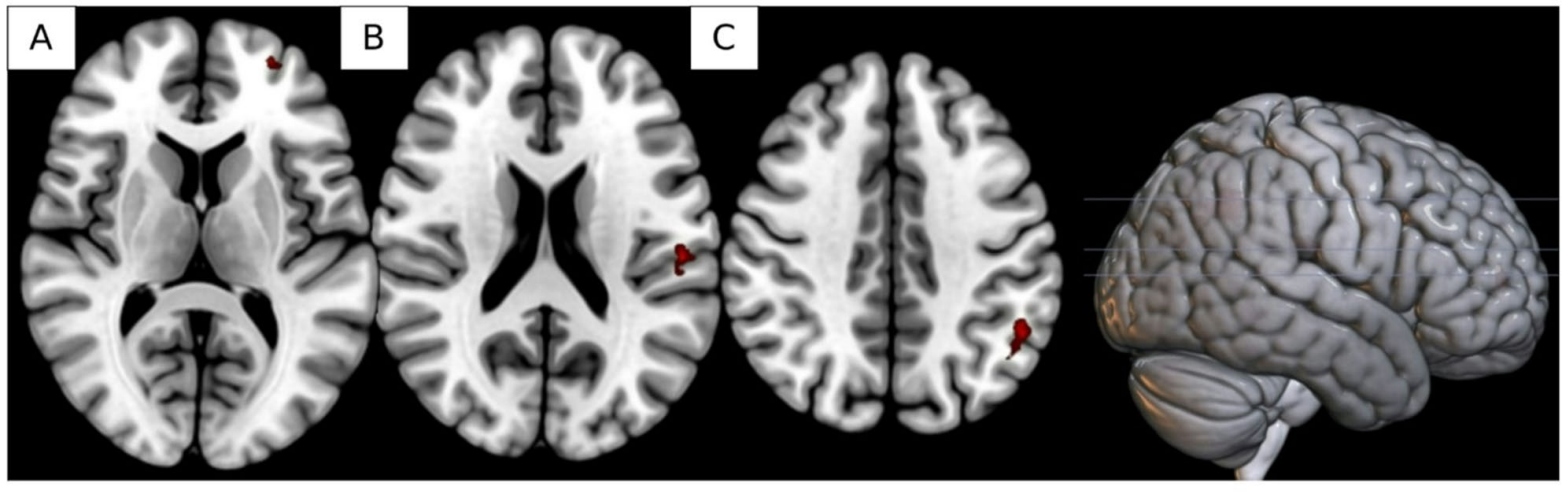
Regions showing significant increases in activation associated with presentation of processed vs. unprocessed food cues from pre- to post-treatment, including (A) left middle frontal gyrus, (B) left supramarginal gyrus, and (C) left angular gyrus.

Figure 4 shows the responses to the HADS Anxiety and Depression scales and the Acceptance and Action Questionnaire. Relative to the screening values, all participants showed reductions in all three measures except for P2’s HADs Anxiety scores. Note there was variability in the absolute values of these measures at screening, see Table 1. At screening, P3, P5, and P6 reported anxiety or depression above the normal range, and P3 and P6 reported levels of psychological inflexibility that might affect well-being (Bond et al., 2011). The decreases across time also reflect some individual variability. P2 showed the least reduction, but this participant also reported low, normal-range screening values, and thus none of P2’s values at the follow-up time points were above the normal range.

Table 3 shows the ratings of clinician and patient global improvement relative to baseline. Most ratings indicated some degree of improvement, from very much (“1”), much improved (“2”), and minimally improved (“3”). Only P2’s ratings reflected no change (“4”) at weeks 4, 6, and 14.

### Anthropometric and biomarker measures

**Fig. 5:**
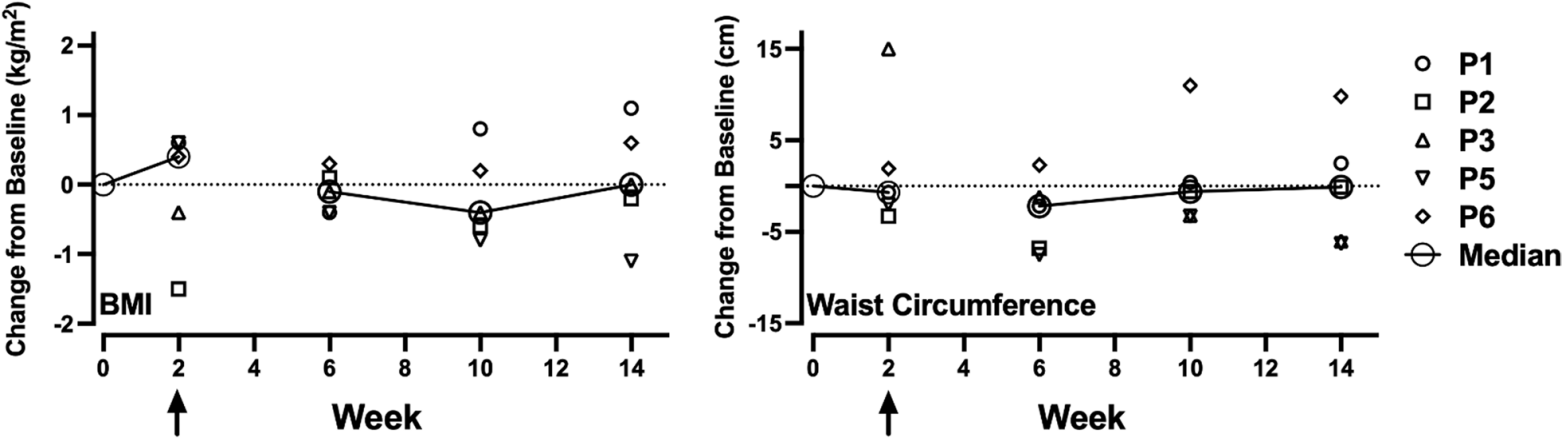
Anthropometric measures across all timepoints. Note: BMI = Body Mass Index. Arrows indicate psilocybin administration

Figure 5 shows changes from screening in BMI (left panel) and waist circumference (right panel). Changes were somewhat variable across time points and participants. At the 14-week follow-up, three participants (P2, P3, and P4) showed reductions in BMI and waist circumference, while the other two showed increases (P1 and P6). No consistent or meaningful changes in the biomarkers were revealed (data in Supplement 2).

### Functional magnetic resonance imaging (fMRI)

Results indicated several clusters where patterns of activation associated with presentation of processed vs. unprocessed food cues changed significantly from pre- to post-treatment. In left middle frontal gyrus (k = 23, -28, 56, 10, pFDR = 0.03; Fig. 4a), left supramarginal gyrus (k = 41, -54, -22, 20, pFDR = 0.002; Fig. 4b), and left angular gyrus (k = 93, -52, -54, 36, pFDR < 0.0001; Fig. 4c), presentation of processed images was associated with lower activation than unprocessed images prior to treatment. Post-treatment, this pattern was reversed. No significant interactions of cue type and session or cue type and fed/fasted state were noted for processed food vs. neutral cues or unprocessed food vs. neutral cues.

## Discussion

This open-label pilot study evaluated the feasibility, safety, and preliminary effects of a single dose of psilocybin-assisted therapy in individuals with binge eating disorder (BED). Psilocybin was well tolerated, with all adverse events rated as mild and resolving without intervention. No serious adverse events or signs of suicidality were observed. Reductions in self-reported binge eating behaviors were evident following treatment and generally sustained through the 14-week follow-up. Improvements were also observed across several domains of self-reported psychological functioning, including reductions in anxiety, depression, and psychological inflexibility. Although changes in anthropometric outcomes (BMI and waist circumference) were variable, a subset of participants demonstrated modest reductions by the final follow-up point.

The current findings converge with a growing body of evidence supporting the safety and potential efficacy of psilocybin-assisted therapy across a range of psychiatric conditions, including substance use disorders, obsessive-compulsive disorder, and anorexia nervosa [8–12, 14–16]. For instance, in a phase 1 open-label trial of psilocybin for anorexia nervosa, participants demonstrated significant reductions in concerns about weight and shape, alongside qualitative reports of increased insight, openness, and cognitive flexibility [15]. These clinical benefits occurred in the absence of significant adverse events, consistent with the safety profile observed in the present study. Similarly, a recent open-label pilot trial in individuals with Parkinson’s disease showed that psilocybin was well tolerated and associated with meaningful improvements in mood and cognitive flexibility [9]. Together, these findings suggest that psilocybin may exert transdiagnostic effects across disorders marked by rigid cognitive, affective, and behavioral patterns, which are core features of BED as well [6].

Subjective responses to psilocybin varied among participants, ranging from minimal psychedelic effects to profound mystical-type experiences. Despite this variability, no clear or consistent association emerged between the intensity of subjective effects and improvements in behavioral or psychological outcomes [54]. Exploratory correlational analyses (not reported) revealed no consistent pattern of associations between either the EBI or MEQ and self-reported eating outcomes; however, given the small sample size, these analyses are best interpreted as descriptive rather than inferential. Nonetheless, this observation aligns with findings from a recent meta-correlation analysis, which estimated that psilocybin-induced subjective effects explain approximately 24% of the variance in therapeutic outcomes across clinical trials in depression and substance use disorders [41]. The analysis found stronger correlations in studies of substance use disorders compared to depression, and noted considerable variability due to differences in study design, measurement timing, and effect quantification. These findings suggest that while subjective effects may play a contributory role, they may be neither necessary nor sufficient predictors of clinical response.

MRI findings indicated significant increases in functional activation within middle frontal gyrus, angular gyrus, and supramarginal gyrus associated with the presentation of processed vs. unprocessed food cues from pre- to post-treatment. These regions are implicated in reward processing, memory, cognitive and inhibitory control, and evaluating stimulus salience [55–59]. Notably, prior studies indicate that individuals with bulimia nervosa and binge eating disorder show reduced lower middle frontal gyrus activity than controls during anticipation and consumption of a highly appetitive food (i.e., a chocolate milkshake) [60]. Thus, although our results are necessarily preliminary given the small sample size and open-label design, future randomized controlled studies should investigate whether increased activity after treatment in this region may reflect a normalization of processing highly appetitive food stimuli. The timing of the post-dose imaging assessment is also an important limitation. Unlike studies that scan within hours or days of psilocybin administration, our follow-up fMRI occurred four weeks after dosing, largely due to logistical constraints. This approach may have missed acute or sub-acute neural effects, though it allowed us to examine more durable changes that may relate to longer-term outcomes. Future studies should include earlier post-dose imaging to capture the full temporal profile of psilocybin’s neural effects.

The present study also examined changes in psychological flexibility, a construct referring to the capacity to remain present and engage in behavior aligned with one’s values despite internal discomfort. Psychological flexibility has been proposed as a transdiagnostic mediator of therapeutic change in psychedelic therapy [32], and recent adaptations of Acceptance and Commitment Therapy have emphasized this process within psychedelic frameworks [37, 38]. In this study, self-reported psychological inflexibility decreased following treatment for all participants except one. Future studies should further examine its role as a putative mechanism of change.

As previously noted, this study is limited by its small sample size, short duration of follow-up, and open-label design, which precludes causal inference and increases the potential for expectancy effects. Additionally, the lack of inferential statistics may limit the generalizability of these findings. The observed heterogeneity in response patterns highlights the need for larger, controlled trials to better characterize moderators of treatment response, including psychological, neural, and experiential factors.

Despite these limitations, this pilot study demonstrates the safety and acceptability of psilocybin-assisted therapy for BED and suggests improvements across behavioral, psychological, and neurophysiological domains are achievable. The convergence of findings across multiple outcome domains supports further investigation of psilocybin in larger, randomized trials to more rigorously test efficacy and elucidate mechanisms of action.

**Table 1:** *Participant demographics and baseline characteristics*

Baseline Characteristic	P1	P2	P3	P5	P6
Age	18	54	55	35	28
Gender	F	M	F	F	F
Race	W	W	W	W	W
Ethnicity	Not Hisp	Not Hisp	Not Hisp	Not Hisp	Not Hisp
Body Mass Index (kg/m^2^)	42.9	44.9	18.7	34.6	35.3
Waist circumference (cm)	106.6	136.3	73	101.9	85.3
Avg daily episodes of large intake	1.8	0.86	1	0.57	0.55
Avg daily loss of control episodes	1.4	0.48	0.75	0.52	0.55
Binge Eating Scale	13	13	37	40	34
Binge + LOC days (past 28 days)	7	4	14	24	4
HADS (Anxiety)	4	4	16	11	11
HADS (Depression)	5	2	4	10	10
Acceptance Action Quest	13	13	38	18	25

**Table 2:** *Physiological*,* behavioral*,* and subjective responses to psilocybin*

Measure	P1	P2	P3	P5	P6
*Cardiovascular measures (peak effects*,* % increase pre-dose)*					
Heart rate (bpm)	88 (29%)	84 (15%)	76 (7%)	85 (27%)	88 (13%)
Systolic BP (mm Hg)	128 (8%)	128 (0%)	155 (37%)	142 (11%)	132 (6%)
Diastolic BP (mm Hg)	80 (25%)	82 (8%)	84 (40%)	101 (20%)	94 (7%)
*Monitor rating scale (peak effects)*					
Overall drug effect	3.5	1	2	3.5	3
Unresponsive to questions	0.5	0	0	0.5	0
Anxiety or fearfulness	1	0.5	1	1.5	1
Distance from ordinary reality	3	0.5	1.5	3	1.5
Ideas of reference/paranoid thinking	0.5	0.5	0	0.5	0.5
Yawning	0.5	0.5	1	1.5	0.5
Tearing/crying	0	0	1.5	3.5	2.5
Nausea/vomiting	0.5	0	0.5	1	0
Visual effects with eyes open	1	0.5	1	1	1
Visual effects with eyes closed	0.5	0.5	2	2.5	1
Spontaneous motor activity	2.5	0.5	1	4	2
Restless/fidgety	2.5	1.5	1	1.5	1.5
Joy/intense happiness	1.5	0	1	2.5	2
Peace/harmony	1.5	0	2	2.5	1.5
Psychological discomfort	0.5	1	1	2	2
Physical discomfort	1	1	0.5	1	0
*Self-report questionnaires (% max total score)*					
Challenging Experiences Quest	8	3	44	13	43
Mystical Experiences Quest	59	8	31	27	80
Emotional Breakthrough Inventory	55	47	13	100	90

**Table 3:** *Clinician and patient global improvement ratings from baseline*

	P1		P2		P3		P5		P6	
Week	CGI	PGI	CGI	PGI	CGI	PGI	CGI	PGI	CGI	PGI
4	-	1	-	4	-	2	-	2	-	2
6	2	1	3	4	2	2	1	1	1	3
10	2	1	2	2	2	3	2	2	1	2
14	2	1	2	4	3	2	2	1	2	2

Note. CGI = Clinician Global Impressions, PGI = Patient Global Impressions

## Supplementary Information

Below is the link to the electronic supplementary material.

Supplementary Material 1

Supplementary Material 2

## Data Availability

The data supporting this study’s findings are available on reasonable request from the corresponding author.
