# Supplementary material for: An open-label pilot study of psilocybin-assisted therapy for binge eating disorder
Source: J Eat Disord. 2026 Jan 3;14:41. doi: 10.1186/s40337-025-01508-3 (PMC12857019; doi:10.1186/s40337-025-01508-3)
Supplement: Supplementary file 1 — Supplementary Material 1 [file 40337_2025_1508_MOESM1_ESM.pdf]

A PHASE 2A SAFETY AND FEASIBILITY STUDY EVALUATING PSILOCYBIN (TRP-8802) ADMINISTRATION IN CONCERT WITH PSYCHOTHERAPY IN THE TREATMENT OF BINGE EATING DISORDER

Protocol Number: TRYP-001

Principal Investigator: Dr. Jennifer Miller

Sponsor: Tryp Therapeutics, Inc

Funded by: Tryp Therapeutics

Version Number: 3.0

03 December 2021

CONFIDENTIALITY/COMPLIANCE STATEMENT

The information in this document contains trade secrets and commercial information that are privileged or confidential to Tryp Therapeutics, Inc. and may not be disclosed unless such disclosure is required by federal or state/province law or regulations. In any event, persons to whom the information is disclosed must be informed that the information is privileged or confidential and may not be further disclosed by them. These restrictions on disclosure will apply equally to all future information supplied to you which is indicated as privileged or confidential.

SIGNATURE PAGE

Protocol Title: A Phase 2a Safety and Feasibility Study Evaluating Psilocybin (TRP-8802) Administration in Concert with Psychotherapy in the Treatment of Binge Eating Disorder

Protocol Number: TRYP-001

Protocol Version: 3.0 (03Dec2021)

Sponsor: Tryp Therapeutics, Inc

Legal Registered Address: **Tryp Therapeutics, Inc.**

301 - 1632 Ellis Street
Kelowna, BC V1Y 2B#
Telephone: 908-727-3312

Manufacturer: Usona Institute

Regulatory Agency Identifier Number(s)

IND: 155844

Approver/Sponsor:

James P. Gilligan, Ph.D., MSIB — Signature James Gilligan

President & CSO, Tryp Therapeutics — Date 12/02/2021

Table of Contents

Investigator's Agreement 5
1 PROTOCOL SUMMARY 6
1.1 Synopsis 6
1.2 Schema 12
1.3 Schedule of Activities (SOA) 13
2 INTRODUCTION 15
2.1 Study Rationale 15
2.2 Background 16
2.3 Risk/Benefit Assessment 17
2.3.1 Known Potential Risks 17
2.3.2 Known Potential Benefits 19
2.3.3 Assessment of Potential Risks and Benefits 19
3 OBJECTIVES AND ENDPOINTS 19
4 STUDY DESIGN 21
4.1 Overall Design 21
4.2 Scientific Rationale for Study Design 21
4.3 Justification for Dose 21
4.4 End of Study Definition 22
5 STUDY POPULATION 22
5.1 Inclusion Criteria 22
5.2 Exclusion Criteria 23
5.3 Screen Failures 24
5.4 Strategies for Recruitment and Retention 24
6 STUDY INTERVENTION 24
6.1 Study Intervention(s) Administration 24
6.1.1 Study Intervention Description 24
6.1.2 Psycohtherapy, Dosing, and Imaging 25
6.2 Preparation/Handling/Storage/Accountability 28
6.2.1 Acquisition and Accountability 28
6.2.2 Formulation, Appearance, Packaging, and Labeling 28
6.2.3 Product Storage and Stability 28
6.3 Measures to Minimize Bias: Randomization and Blinding 28
6.4 Study Intervention Compliance 28
6.5 Concomitant Therapy 28
7 STUDY INTERVENTION DISCONTINUATION AND PARTICIPANT DISCONTINUATION/WITHDRAWAL 28
7.1 Discontinuation of Study Intervention 28
7.2 Participant Discontinuation/Withdrawal from the Study 29
7.3 Lost to Follow-Up 29
8 STUDY ASSESSMENTS AND PROCEDURES 30
8.1 Clinical Activity Assessments 30
8.2 Safety Assessments 32
8.3 Adverse Events and Serious Adverse Events 32
8.3.1 Definition of Adverse Events (AE) 32
8.3.2 Definition of Serious Adverse Events (SAE) 32
8.3.3 Classification of an Adverse Event 33
8.3.4 Time Period and Frequency for Event Assessment and Follow-Up 35

8.3.5 Adverse Event Reporting 35
8.3.6 Serious Adverse Event Reporting 35
8.3.7 Reporting Events to Participants 36
8.3.8 Reporting of Pregnancy 36
9 STATISTICAL CONSIDERATIONS 36
9.1 Statistical Hypotheses 36
9.2 Sample Size Determination 36
9.3 Populations for Analyses 37
9.4 Statistical Analyses 37
9.4.1 General Approach 37
9.4.2 Analysis of the Primary Endpoint(s) 37
9.4.3 Analysis of the Secondary Endpoint(s) 38
9.4.4 Safety Analyses 39
9.4.5 Baseline Descriptive Statistics 39
9.4.6 Planned Interim Analyses 39
9.4.7 Sub-Group Analyses 40
9.4.8 Tabulation of Individual participant Data 40
9.4.9 Exploratory Analyses 40
10 SUPPORTING DOCUMENTATION AND OPERATIONAL CONSIDERATIONS 40
10.1 Regulatory, Ethical, and Study Oversight Considerations 40
10.1.1 Informed Consent Process 40
10.1.2 Study Discontinuation and Closure 40
10.1.3 Confidentiality and Privacy 41
10.1.4 Key Roles and Study Governance 42
10.1.5 Safety Oversight 42
10.1.6 Clinical Monitoring 43
10.1.7 Quality Assurance and Quality Control 44
10.1.8 Data Handling and Record Keeping 45
10.1.9 Protocol Deviations 45
10.1.10 Publication and Data Sharing Policy 46
10.1.11 Conflict of Interest Policy 46
10.2 Abbreviations 48
Appendix A: Dosing and Therapy Sessions 50
11 REFERENCES 52

I, the undersigned, am responsible for the conduct of the study at my site and agree to the following:

- I understand and will conduct the study in accordance with the approved study protocol and its attachments, any approved protocol amendments, all statements of confidentiality, Good Clinical Practices, International Conference on Harmonisation Good Clinical Practice (ICH GCP) and applicable United States (US) Code of Federal Regulations (CFR).
- I will not deviate from the study protocol without prior written permission of the Sponsor or its designee and prior written approval from the Institutional Review Board or Independent Ethics Committee (if applicable), except where necessary to prevent any immediate danger to the subject.
- I will ensure that the protocol, informed consent form(s), recruitment materials, and all participant materials are submitted to the Institutional Review Board (IRB) for review and approval. Approval of both the protocol and the consent form must be obtained before any participant is enrolled. Any amendment to the protocol requires review and approval by the IRB before the changes are implemented to the study. All changes to the consent form will be IRB approved; a determination will be made regarding whether a new consent needs to be obtained from participants who provided consent, using a previously approved consent form.
- I have read and understand fully the current version of the Investigator's Brochure (IB) for Psilocybin [3-[2-(dimethylamino) ethyl]-1H-indol-4-yl] dihydrogenphosphate.
- I have sufficient time, an adequate number of qualified staff, and adequate facilities to conduct and complete the study according to the protocol, properly, safely and within the anticipated timeline.
- I will ensure that all staff at my site who are involved in the study are adequately trained regarding the investigational products, the study protocol, GCP and related regulations, and their responsibilities. In case of delegation of any of my study responsibilities, I will ensure that I delegate to qualified staff and this information is captured in a log or other form of documentation for the study files.

[illegible] 12-3-2021
Signature of Investigator | Date

Jennifer Miller MD | University of Florida
Printed Name | Site Number and Institution

1 PROTOCOL SUMMARY

1.1 SYNOPSIS

**Title:**	TRYP-001: A Phase 2a Safety and Feasibility Study Evaluating Psilocybin (TRP-8802) Administration in Concert with Psychotherapy in the Treatment of Binge Eating Disorder
**Primary Objective:**	The primary objective of this study is to:<br>1. Assess the safety of a single dose of TRP-8802 in participants with binge eating disorder (BED) during the TRP-8802 dosing session, and through 12 weeks following dosing (i.e., Week 14).
**Secondary and Exploratory Objectives:**	The secondary objectives of this study are to:<br>1. Evaluate the feasibility of inducing the psychedelic state with TRP-8802 in a BED population.<br>2. Determine the preliminary clinical activity and the effects of TRP-8802 in conjunction with psychotherapy on the frequency of binge-eating episodes and other weight-related indicators in a BED population through 4 weeks following dosing (i.e., Week 6).<br>The exploratory objectives of this study are to:<br>1. Determine the effects of TRP-8802 on multiple ancillary indicators of clinical activity in a BED population throughout the study until 12 weeks following dosing (i.e., Week 14).<br>2. Assess the relationship between clinical activity and the intensity of the psychedelic experience.
**Rationale:**	Binge eating disorder is the most common eating disorder and is associated with obesity and psychiatric comorbidities, including depression, and impulsive and compulsive disorders. Binge eating disorder is marked by severe disturbance to a person's control over their eating behaviors and high anxiety around food. Various programs using psilocybin paired with psychotherapy have shown positive effects in treating a variety of psychiatric and behavioral conditions, including cancer-related psychiatric distress, anxiety, treatment-resistant depression, and nicotine and alcohol addiction. Based on clinical precedents, relevant neuropharmacology, and mechanistic similarities, psilocybin is theorized to have the potential to be part of the treatment of overeating disorders. TRP-8802 could accomplish this by moderating overall anxiety, anxiety around food, perseveration, and repetitive and intrusive thoughts about food in people with BED.
**Endpoints:**	The primary endpoint of the trial is:<br>1. Nature and severity of adverse events (AEs); changes in vital signs, physical examination, electrocardiogram (ECG), clinical laboratory parameters, Columbia-Suicide Severity Rating Scale (C-SSRS) through 12 weeks following dosing.<br>The secondary endpoints of the study are:<br>1. Magnitude and duration of TRP-8802-induced dissociative effects in

	participants with BED using the Mystical Experience Questionnaire-30 item (MEQ30) and Monitor Rating Scale (MRS).<br>2. CFB through 4 weeks following TRP-8802 dosing in:<br>i. Frequency of binge eating episodes<br>ii. Clinical Global Impression-Improvement (CGI-I) scale<br>iii. Waist circumference<br>iv. Body mass index (BMI)<br>The exploratory clinical activity endpoints of this study are to:<br>1. Determine the effects of TRP-8802 on multiple ancillary outcomes in adults with BED, including CFB at specified time points through 12 weeks following TRP-8802 dosing.<br>2. Assess the relationship between clinical activity and the intensity of the psychedelic experience, as measured by MEQ30, the Challenging Experiences Questionnaire (CEQ), Emotional Breakthrough Inventory (EBI), and MRS.
**Design:**	Phase 2a Open Label Safety and Feasibility Study
**Population:**	Adults with BED
**Anticipated No. of:**	Subjects: 10<br>Sites: 1<br>Countries: US
**Inclusion/Exclusion Criteria:**	Inclusion Criteria:<br>To participate in this study, subjects will have to meet all of the following criteria:<br>1. Meet Diagnostic and Statistical Manual of Mental Disorders, 5th Edition (DSM-5) criteria for BED.<br>2. Age ≥18 and ≤64 years.<br>3. Provision of signed and dated informed consent form.<br>4. Stated willingness to comply with all study procedures and availability for the duration of the study.<br>5. Medically stable in the judgment of the Principal Investigator, as determined by screening medical, physical examination, ECG, and routine laboratory tests including blood and urinalysis.<br>6. For females of reproductive potential: use of highly effective contraception for at least 1 month prior to screening and agreement to use such a method during study participation and for an additional 4 weeks following the dose of TRP-8802. Adequate birth control methods include intrauterine device; injected, implanted, intravaginal, or transdermal hormonal method; oral hormones plus a barrier contraception; abstinence; vasectomized sole partner; or double barrier contraception.<br>7. For males of reproductive potential: use of condoms or other methods to ensure effective contraception with partner through 90 days post-dose.

8. Agree to consume approximately the same amount of caffeine-containing beverage (e.g., coffee, tea) that he/she consumes on a usual morning, before arriving at the research unit on the morning of the drug session day. If the participant does not routinely consume caffeinated beverages, he/she must agree not to do so on the dosing session day.
9. Agree to refrain from using any psychoactive drugs, including alcoholic beverages for a minimum of 1 week prior to drug administration.
10. Agree that for 1 week before the drug session, including the morning of the session, he/she will refrain from taking any nonprescription medication, nutritional supplement, herbal supplement, or as needed (PRN) prescription medication except when approved by the study investigators. Exceptions will be evaluated by the study investigators and will include acetaminophen, non-steroidal anti-inflammatory drugs, and common doses of vitamins and minerals and contraceptives.

Exclusion Criteria:

To participate in this study, subjects must not meet any of the following criteria:

1. Significant suicide risk as defined by either suicidal ideation as endorsed on items 4 or 5 on the C-SSRS within the past year, at Screening, or at Baseline; or suicidal behaviors within the past year; clinical assessment of significant suicidal risk during subject interview.
2. Participation in another concurrent clinical study or within the preceding month.
3. Women who are pregnant or who intend to become pregnant during the study or who are currently nursing.
4. Vital signs, averaged over 3 readings within 15 minutes, of systolic blood pressure (BP) >139 mm Hg, diastolic BP >89 mm Hg, or heart rate >90 bpm.
5. Have any of the following cardiovascular conditions: uncontrolled hypertension, coronary artery disease, congenital long QT syndrome, cardiac hypertrophy, cardiac ischemia, congestive heart failure, prior myocardial infarction, tachycardia, artificial heart valve, QTc >450 msec at screening, any other clinically significant screening ECG abnormality, or any other significant cardiovascular condition.
6. Presence of a gastrointestinal disease that could interfere with absorption of orally-administered TRP-8802.
7. Have epilepsy.
8. Meet DSM-5 criteria for schizophrenia spectrum or other psychotic disorders, including major depressive disorder with psychotic features, or Bipolar I or Bipolar II Disorder.
9. Family history of psychosis.
10. Meet DSM-5 criteria for a moderate or severe alcohol or drug use disorder.

	11. Positive urine drug screen or alcohol breath test at screening. A repeat test can be conducted at screening or Day –1 at the discretion of the Principal Investigator or delegate.<br>12. Prior adverse effects from psilocybin.<br>13. Currently taking or expected to need prior to the dosing session, UGT1A9 or 1A10 inhibitors (e.g., regorafenib, rifampicin, phenytoin, eltrombopag, mefenamic acid, diflunisal, niflumic acid, sorafenib, isavuconazole, deferasirox, ginseng) and aldehyde or alcohol dehydrogenase inhibitor (e.g., disulfiram).<br>14. Currently taking or testing positive on urine drug screen, drugs of abuse such as amphetamines, buprenorphine, benzodiazepines, cocaine, methamphetamines, Ecstasy (MDMA), morphine, methadone, oxycodone, marijuana, ethyl glucuronide, fentanyl, tramadol, and synthetic cannabinoids (K2).<br>15. Currently taking on a regular (e.g., daily) basis any medications having a primary centrally-acting serotonergic effect, including SSRIs, MAOIs, or serotonin-acting dietary supplements (such as 5-hydroxy tryptophan or St. John's wort). For individuals who have intermittent or PRN use of such medications, the dosing session will not be conducted until at least 5 half-lives of the agent have elapsed after the last dose.<br>16. fMRI subjects: Contraindications to fMRI procedures, per institutional policy.
**Treatment:**	A single (1) 25 mg oral dose of TRP-8802 will be administered in a carefully monitored setting following 6 to 8 hours of preparatory psychotherapy.
**Concomitant Medications:**	Medications which are not specifically excluded are allowed, unless the Principal Investigator (PI) or Medical Monitor deems that use of the medications will interfere with interpreting the results of the study.<br>All concomitant medications will be recorded in the electronic case report form (eCRF).
**Visit Summary:**	Screening:<br>Subjects will attend at least 1 visit for screening. The screening period must be completed before the Run-in period. This period will include hematology, blood chemistry, urine drug screen and other safety assessments to evaluate medical inclusion criteria. Assessments conducted during Screening will be considered baseline values for the purpose of determining CFB.<br><br>Run-in:<br>Assessment of daily binge eating episodes for the 2 weeks during Run-in will be considered baseline values for the purpose of determining CFB in binge eating frequency.<br><br>Baseline:<br>fMRI and electroencephalogram (EEG) measures conducted during the 1-week

	Baseline period will be considered baseline values for the purpose of determining CFB.<br><br>Visits related to medication dosing:<br><br>To enhance participant safety, a Set and Setting protocol will be utilized similar to the protocol that has been used in all modern studies of psilocybin. These sessions are described in detail in Section 6.1.2 and in Appendix A: Dosing and Therapy Sessions. The Set and Setting protocol for this study includes: 1) two preparation sessions with therapists prior to dosing; 2) administration of study medication in an aesthetically pleasing room under the supervision of the same pair of therapists who are present throughout the session; 3) two integration sessions after dosing (day after dose and ~7 days after dose); and, 4) a follow-up session to coordinate study termination and plan for long-term maintenance of gains. The PI may join the therapist for the preparatory and post-dose integration sessions. The same pair of therapists will follow individual participants throughout their preparatory, dosing, and integration study experience.<br><br>Other assessments processes:<br><br>Subjects will have telephone follow-up visits for safety at 2, 3, and 6, and 10 weeks post dosing; and in-person follow-up visits at 4 (secondary endpoint assessment), 8, and 12 weeks post dose. The detailed schedule is provided in the schedule of activities (SOA).
**Study Duration:**	Subjects will be in the study for 12 weeks following the dose of TRP-8802 (until Week 14), approximately 5 months from initiation of screening through the last follow-up.
**Clinical Activity and Feasibility Assessments:**	All rating scales and other clinical activity assessments are described in endpoints above.
**Safety:**	Nature and severity of AEs will be recorded throughout the study. During dosing sessions, manifestations of the psychedelic experience will not be recorded as AEs, unless judged by the monitors to exceed the intensity and/or duration of expected reactions.<br><br>In-session safety:<br>• Vital signs (heart rate and BP) will be assessed before capsule administration and at 30, 60, 90, 120, 180, 240, 300, and 360 minutes after capsule administration.<br>• 12-lead ECGs will be performed in triplicate pre-dose and 60, 120, and 360 minutes after capsule administration.<br>• C-SSRS and AEs: Pre- and post-dosing session<br><br>Day after dosage safety:<br>• Physical examination, vital signs, blood chemistry and hematology laboratory assessments, C-SSRS and AEs<br><br>Post-dose follow-up safety:

	• The PI will have a safety check phone call with participants 2, 3, 6, and 10 weeks post dose to collect C-SSRS and AEs<br>• Post-dose clinic visits, 4, 8, and 12 weeks post dose, to monitor vital signs (heart rate and BP), ECG, blood chemistry and hematology laboratory assessments, C-SSRS, and AEs
**Safety Review Committee:**	The Safety Review Committee (SRC) will be responsible for the assessment of safety and to make decisions with regards to study progression. The SRC will be composed of at least the PI, the study's Principal Psychologist, one medically qualified Sponsor representative (SRC Chair), and a biomedical professional with relevant experience and expertise. The SRC is responsible for reviewing study procedures, enrollment, drop-outs, and safety data such as AEs, vital signs, laboratory tests, ECGs, etc. The SRC members can ask questions and make comments and/or recommendations to the Sponsor.
**Analysis Populations:**	Safety Population: all participants for whom the experimental procedure is initialized. Participants who do not receive any amount of the study drug will still contribute to the safety analysis if the procedure was initialized but not completed, since all AEs of administration must be assessed. All safety analyses will utilize the Safety Population.<br>Full Analysis Set (FAS): all participants who receive the single dose of study drug, and who have any post-baseline clinical assessments. The primary and secondary endpoint analyses will be performed using data from the FAS Population.<br>Per Protocol (PP): a subset of the FAS population, those who complete 4 weeks of follow up post dosing with no major protocol deviations as determined by a review of participant data. The analysis of clinical activity based on the PP population will be considered supportive of the FAS analysis.
**Data Analyses:**	Data will be handled and processed according to CRO's Standard Operating Procedures (SOPs), which are written based on the principles of Good Clinical Practices and Good Clinical Data Management Practices.<br>Detailed methodology for summary and statistical analyses of the data collected in this study will be documented in a Statistical Analysis Plan (SAP) which will be finalized prior to study completion. The SAP may modify the plans outlined in the protocol; however, any major modifications of the primary endpoint definition and/or its analysis will also be reflected in a protocol amendment. Additional statistical analyses other than those described in the protocol may be performed if deemed appropriate and included in the SAP.

1.2 SCHEMA

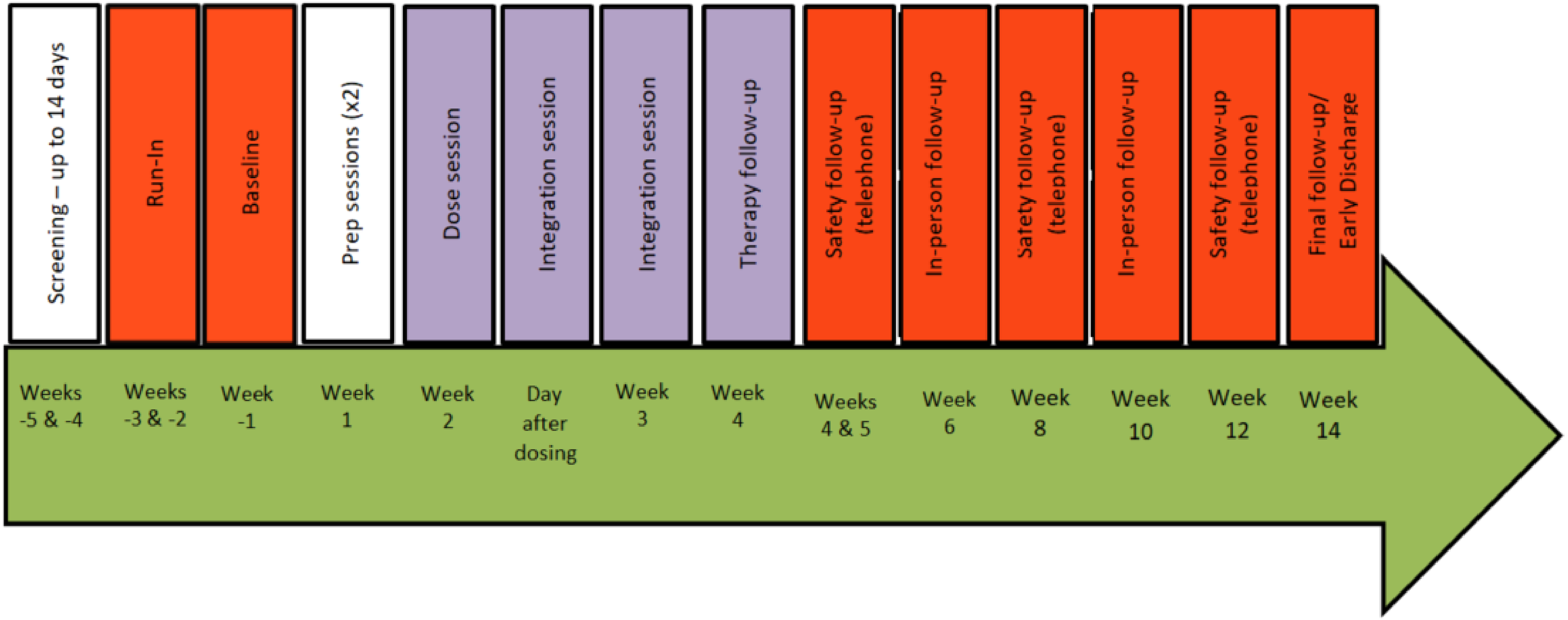
Screening – up to 14 days
Run-In
Baseline
Prep sessions (x2)
Dose session
Integration session
Integration session
Therapy follow-up
Safety follow-up (telephone)
In-person follow-up
Safety follow-up (telephone)
In-person follow-up
Safety follow-up (telephone)
Final follow-up/ Early Discharge
Weeks -5 & -4
Weeks -3 & -2
Week -1
Week 1
Week 2
Day after dosing
Week 3
Week 4
Weeks 4 & 5
Week 6
Week 8
Week 10
Week 12
Week 14

1.3 SCHEDULE OF ACTIVITIES (SOA)

Event/ Procedure	Screening	Run-in	Baseline	Prep Sessions 1 & 2 [1]	Medication Dosing Day [1]	Integration Session 1 [1]	Integration Session 2 [1]	Therapy Follow-up [1]	Safety Follow-up	Follow-up/ Early Discharge
Timing Windows of ±2 days	Weeks -5 and -4	Weeks -3 and -2	Week -1	Week 1	Week 2	Day after dose	Week 3	Week 4	Weeks 4, 5, 8, 12	Weeks 6, 10, 14
Visit type	In-person	Remote	In-person	Video/ In-person	In Unit	In-person	Video/ In-person	Video/ In-person	Telephone	In-person
Informed Consent	X									
Psychiatric History [2]	X									
Medication History	X									
Entry Criteria	X									
Vital Signs [3]	X				X	X				X
12-lead ECG [4]	X				X	X				X
Physical Exam	X					X				X
Height, Weight, BMI	X				X					X
Waist circumference	X				X					X
Chemistry [5], hematology [6]	X				X	X				X
Urinalysis [7], drug screen [8]	X				X [9]					
Alcohol breath test	X				X [9]					
Pregnancy test [10]	X				X					
Therapy session [11]				X		X	X	X		
fMRI and EEG			X							X [12]
Administer oral TRP-8802					X					
C-SSRS [13]	X		X		X	X	X	X	X	X
MEQ30						X				
CEQ						X				
EBI						X				
MRS					X [14]					
Metabolic biomarkers					X					X
BES	X									X

Event/ Procedure	Screening	Run-in	Baseline	Prep Sessions 1 & 2 [1]	Medication Dosing Day [1]	Integration Session 1 [1]	Integration Session 2 [1]	Therapy Follow-up [1]	Safety Follow-up	Follow-up/ Early Discharge
Timing Windows of ±2 days	Weeks -5 and -4	Weeks -3 and -2	Week -1	Week 1	Week 2	Day after dose	Week 3	Week 4	Weeks 4, 5, 8, 12	Weeks 6, 10, 14
Binge eating (modified Eating Questionnaire, daily)		X	X	X		X	X		X [15]	X [15]
Binge eating (Eating Questionnaire, q 4 wks)	X [16]									X [17]
CGI-I	X									X
PGI-I	X							X		X
HADS [13]	X							X		X
AAQ-II	X							X		X
Record adverse events	X [18]	X	X	X	X [19]	X	X		X	X
Concomitant medications	X		X	X	X	X	X		X	X

[1] Described in Appendix A.
[2] Psychiatric history assessed by PI and study psychologist.
[3] Full seated vital signs (heart rate and blood pressure) at screening and upon admission to clinic on dosing day. Brief vital signs will be assessed before capsule administration and at 30, 60, 90, 120, 180, 240, 300, and 360 minutes after capsule administration and the following day. Brief seated vital signs at each in-person follow up visit.
[4] 12-lead ECG in triplicate at screening, pre-dose and 60, 120, and 360 minutes after capsule administration. ECGs will also be obtained for early terminators.
[5] Chemistry panel (Na+, K+, Cl-, HCO3-, Ca++, Mg++, P, BUN, creatinine, glucose, total bilirubin, albumin, ALT, AST, GGT, CK, LDH, alkaline phosphatase) on dosing day prior to dosing.
[6] CBC with white cell differential and platelet count on dosing day prior to dosing.
[7] Urinalysis includes pH, specific gravity, protein, occult blood, glucose, ketones; plus microscopic examination of sediment for RBC, WBC, epithelial cells, casts, crystals and bacteria.
[8] Urine drug screen to be standard 14 (AMP, BUP, BZO, COC, mAMP, MDMA, MOP, MTD, OXY, THC, ETG, FTY, TRA, K2).
[9] Urine drug screen and alcohol breath test performed prior to dosing on dosing day.
[10] Pregnancy test for women of reproductive potential. Test performed prior to dosing on dosing day.
[11] Further details on therapy sessions are described in Appendix A: Dosing and Therapy Sessions.
[12] fMRI and EEG done at Week 6 visit only.
[13] On dosing day, complete prior to dosing and prior to discharge. During follow up, if there are psychiatric concerns, C-SSRS can be done weekly along with HADS.
[14] Conducted at same time as vital signs during dosing session.
[15] Completed daily through Weeks 4, 5, and 6 only.
[16] Completed once for the 4 weeks prior to Screening
[17] Completed once at Weeks 10 and 14 only.
[18] Medical occurrences that begin before the Baseline visit but after obtaining informed consent will be recorded as medical history, not as adverse events.
[19] On dosing day, complete prior to dosing and prior to discharge.

2 INTRODUCTION

2.1 STUDY RATIONALE

Binge eating disorder (BED) is the most common eating disorder and is associated with obesity and psychiatric comorbidities, including depression (*Guerdjikova et al., 2021*). BED is characterized by recurrent episodes of excessive food intake accompanied by a sense of loss of control and psychological distress but without the inappropriate compensatory weight loss behaviors of bulimia nervosa (*Guerdjikova et al., 2021*; *McElroy et al., 2015*). BED is associated with abnormal neural responses to food, especially highly palatable foods (*Citrome, 2019*; *Donnelly et al., 2018*; *Boswell et al., 2021*). Additionally, individuals affected by this condition suffer from severe anxiety and can have significant compulsive and impulsive behaviors, particularly around eating (*Guerdjikova et al., 2019*; *Samodien & Chellan, 2021*). Self-mutilatory behaviors, such as skin picking can be seen in BED, presumably associated with abnormal neuronal connectivity (*Houazene et al., 2021*). Therapeutic agents that influence reward and executive function systems are believed to have potential for treatment of BED (*Boswell et al., 2021*). Because psilocybin alters neuronal connections, the potential opportunities of this therapy include treatment of overall anxiety, anxiety around food, compulsive and impulsive behaviors, self-mutilatory behaviors, and repetitive and intrusive thoughts, especially about food in people with BED.

Variations on psilocybin paired with psychotherapy have shown some evidence of effectiveness in treating cancer-related psychiatric stress, depression and anxiety, and nicotine and alcohol addiction. Clinical trials are ongoing to evaluate the efficacy of psychedelics for anorexia nervosa (NCT04052568, NCT04661514, NCT04505189), a condition where intrusive thoughts drive maladaptive and life-threatening behavior and in which treatment resistance is common (*Foldi et al., 2020*). Opportunities for the use of psilocybin to treat hyperphagic disorders include treatment of anxiety around food, perseveration, and repetitive and intrusive thoughts, especially around food. There are parallels between intrusive thoughts in people with addiction, anorexia nervosa/binge eating and obsessive compulsive disorder (OCD), where affected individuals ruminate on the potential relief afforded by food, often devising elaborate plans to get food (*Lutter & Nestler, 2009*). Positive effects of psychedelics have been seen on depression and wellbeing scores in individuals reporting an eating disorder (*Spriggs et al., 2021*).

The action of psilocybin has been primarily associated with activation of the 5-HT2a receptor. The 5-HT2a receptor has been associated with hyperphagia in animal studies and in some human studies. Stimulation of 5-HT2A receptors in the paraventricular hypothalamus attenuates neuropeptide Y-induced hyperphagia through activation of corticotropin releasing factor (*Grignaschi et al., 1996*). The 5-HT2C/2B receptor agonist m-chlorophenylpiperazine inhibited 2-deoxy-D-glucose-induced hyperphagia in rats (*Sugimoto et al., 2001*). The association of 5-HT2a receptor gene polymorphism with eating disorders has also shown some

conflicting results (*Serretti et al., 2007*). Studies using positron emission tomography and single photon emission computed tomography with 5-HT-specific radioligands have consistently shown 5-HT(1A) and 5-HT(2A) receptor and 5-HT transporter alterations in anorexia nervosa and bulimia in cortical and limbic structures, which may be related to anxiety, behavioral inhibition, and body image distortions (*Bailer & Kaye, 2010*). Overweight was found to be associated with increased 5-HT2A binding in most cortical regions in humans (*Erritzoe et al., 2009*). Ayahuasca, which contains the psychoactive component N,N-dimethyltryptamine which is a 5-HT2a agonist, has shown some positive effects on eating disorder related thoughts and symptoms (*Lafrance et al., 2017*; *Renelli et al., 2020*).

To better understand the potential benefits of psychedelics in overeating disorders, Tryp Therapeutics will conduct a safety and feasibility clinical trial using TRP-8802 among individuals with BED. Key procedures will be adapted from other clinical trials using psilocybin, including psychotherapy sessions for participants to both prepare for and to integrate the psilocybin experience after administration *(Davis et al., 2021*) and implementing standard safety procedures for human psychedelic research (*Johnson et al., 2008*).

2.2 BACKGROUND

Psilocybin (3-[2-(dimethylamino) ethyl]-1H-indol-4-yl] dihydrogen phosphate) is a natural product produced by numerous species of Psilocybe mushrooms and is manufactured for clinical use to control potency and purity. Psilocybin is a tryptamine derivative, and in humans the phosphate group is rapidly enzymatically cleaved in the body to produce psilocin, an agonist at a variety of serotonin receptors, the most important of which in this setting is the 5-HT2A receptor (*Carhart-Harris et al., 2014*; *Nichols, 2004*). Oral psilocybin has about a 50% bioavailability, and psilocin is detectable in plasma within 20 minutes of administration of the parent compound (*Brown et al., 2017*; *Hasler et al., 1997*). The half-life of psilocin in blood is 2to 3 hours. Onset of noticeable psychoactive effects occurs within 1 hour, peaks at about 2 hours after a dose, and loss occurs typically around 6 hours after the dose. Based on this time course, protocols mandate observation in the clinical trial setting until 8 hours after dosing. Further, exposure following a 25 mg oral dose is associated with both near-maximal occupancy of neocortical serotonin 5-HT2A receptors and subjective intensity ratings of elements of the psychedelic experience that have been repeatedly associated with longer-term therapeutic benefits (*Madsen et al., 2019*).

Psilocybin reliably induces profound changes in sensory perception, emotion, thought, and sense of self, which are characterized by marked alterations in all mental functions, including perception, mood, volition, cognition and self-experience (*Geyer & Vollenweider, 2008; Studerus et al., 2011*). These profound changes are often referred to as mystical-type experiences. Measures of mystical-type experience occurring during psilocybin treatment have been repeatedly observed to predict later effects on behavior and emotions, including

reductions in depressive and anxious symptoms (*Griffiths et al., 2016*; *Maclean et al., 2011*; *Ross et al., 2016*).

Non-clinical in vivo and in vitro studies, found via literature searches, demonstrate that, similar to humans, when psilocybin is administered orally to rats it is rapidly dephosphorylated to psilocin in the intestinal mucosa by alkaline phosphatase and a nonspecific esterase, with approximately 50% of the total volume of psilocin absorbed from the digestive tract (*Kalberer et al., 1962*). Maximum plasma levels are achieved after approximately 90 minutes (*Chen et al., 2011*). When administered systemically (i.e., bypassing the gut), initial psilocybin metabolism is performed by tissue phosphatases, with in vitro studies indicating the kidneys as being among the most active metabolic organs (*Horita & Weber, 1961*). Across species tested, the highest levels of psilocin were found in the neocortex, hippocampus, and thalamus (*Hopf & Eckert, 1974*).

Recent clinical studies utilizing pharmaceutical-grade oral psilocybin under controlled conditions have been performed in healthy volunteers and various subpopulations to characterize the safety profile and evaluate the biological response of orally administered psilocybin. Though the safety reporting criteria and the level of data verification varied greatly between studies, including many participant-reported outcomes, these data have been utilized to elucidate the expected adverse event (AE) profile of TRP-8802. The clinical studies to date show similar safety profiles, with both psychologicaland physical AEs reported. The most common adverse psychological events included anxiety, negative emotional states and paranoid/delusional thinking during dosing sessions, and the most common physical effects were increased blood pressure (BP) and heart rate, mild nausea, and mild headache.

Preliminary efficacy of psilocybin in clinical studies showed a decrease in symptomatic response in indications including OCD, substance use disorder, depression, and anxiety. Overall, psilocybin has been well tolerated at the doses examined in the clinical studies which are comparable to the planned dose of 25 mg of TRP-8802 in the current study (*Tryp IB, 2021 v2.1*). Due to the psychoactive nature of the compound, it should only be administered in a controlled settingand per the accompanying clinical protocol.

2.3 RISK/BENEFIT ASSESSMENT

2.3.1 KNOWN POTENTIAL RISKS

Thousands of participants have received psilocybin under controlled conditions in a clinical setting for various indications, with subsequent results published in peer-reviewed journals (*Metzner, 2005*; *Rucker et al., 2018)*. As these studies were predominantly performed in an academicsetting, safety reporting criteria and the level of data verification varied greatly between studies, but these data can be utilized to elucidate the expected AE profile of TRP-8802. Overall, the most commonly reported AEs associated with psilocybin administration are psychological in nature and include anxiety, the induction of negative emotional states and

paranoid/delusional thinking during psilocybin sessions, as well as far less frequent reports of Hallucinogen Persisting Perception Disorder (HPPD) (*Johnson et al., 2008*; *Tylš et al., 2014*). Rates of prolonged psychiatric symptoms of any kind following psilocybin exposure in healthy study participants are estimated to be 0.08-0.09%. Cardiovascular changes including increased BP and heart rate, nausea, and headaches are also commonly reported with psilocybin administration.

Hallucinogen Persisting Perception Disorder (HPPD): Some people who have used serotonergic hallucinogens, such as psilocybin, experience persistent, distressing alterations in mostly visual perception that last from weeks to years after use (*Espiard et al., 2005*). This condition is now diagnosed as HPPD. To date, however, no cases of HPPD have occurred in volunteers given psilocybin in current research studies (*Studerus et al., 2011*). In studies involving cancer patients examining cancer related anxiety and depression, no cases of HPPD were identified and no participants developed any symptoms of paranoia or anxiety that required pharmacological intervention or anything more than reassurance from session therapists. The risk of HPPD occurring after psilocybin administration can be reduced by screening participants for potential risk factors such as substance dependence and by excluding people reporting HPPD or other significant AEs after prior use of hallucinogens.

QTc Interval: Psilocybin doses ranging from 0.3 to 0.6 mg/kg (corresponding to absolute doses of 19 to 59 mg)resulted in a positive effect on QTc prolongation with a linear relationship between maximum plasma psilocin concentration and ΔQTcF (*Dahmane et al., 2021)*. No delay between the time course of psilocin PK and the change in ΔQTcF was observed; i.e., the maximum ΔQTcF was observed at the time of psilocin C_{max}. At the therapeutic dose of 25 mg, the expected mean psilocin C_{max} is about 18.7 ng/mL and the associated upper bound of the 90% CI of the predicted mean ΔQTcF is 6.6 msec, below the threshold of 10 msec. The concentration-QTc analysis by Dahmane and colleagues demonstrated that at a psilocin C_{max} of 60 ng/ml, which is approximately 3 times higher than the expected C_{max} following an oral psilocybin dose of 25mg, ΔQTcF remains below 10 msec with a mean predicted ΔQTcF of 9.1 msec and a 90% upper CI limit for mean ΔQTcF of 17.9 msec. These observations suggest that there is a limited potential of QTc prolongation under psilocybin at the intended investigational dose of 25 mg but that this should be followed during clinical evaluations.

Blood Pressure: Higher doses of psilocybin (>0.3 mg/kg) also may transiently lead to elevated mean BP, peaking 30-60 min following psilocybin administration and returning to baseline levels after 90-180 min without necessitating further interventions (*Griffiths et al., 2006*; *Hasler et al., 2004*). The severity of elevations in BP were usually asymptomatic and were graded as mild or moderate (CTCAE Grade 1 or 2, respectively). Although several subjects in the University of Wisconsin dose escalation study reached BP elevations that were graded as moderate, they remained asymptomatic (Brown et al., 2017). It is not clear whether the changes in BP and heart rate are due to the elevated psilocin concentration directly or to the psychedelic effect caused by this active metabolite. Psilocybin appears to produce only slight sympathetic system

activation. Psilocybin may elevate prolactin, but not cortisol or ACTH (*Gouzoulis-Mayfrank et al., 1999*) with prolactin elevation no longer detectable 300 minutes post-drug (*Hasler et al., 2004*).

Heart Rate: Transient elevations of heart rate are common in subjects receiving doses of psilocybin at doses of 0.3 mg/kg or more. The time course of these elevations in heart rate are similar to those seen for the elevation in BP, peaking between 60-120 minutes after the dose. This is similar to time of peak psilocin concentrations and peak psychedelic effect. Again, it is not clear whether or not the changes in BP and heart rate are directly due to the elevated psilocin concentration or caused indirectly by the psychedelic effect. In a Phase 1 dose-escalation study in healthy volunteers, there were several instances in which mild bradycardia and tachycardia was noted. In a retrospective analysis, it was demonstrated that psilocybin resulted in an increased heart rate, with slightly higher mean change from baseline in heart rate (ΔHR) at higher psilocybin doses and corresponding higher psilocin exposures (*Dahmane et al., 2021*). The maximum mean ΔHR was observed at the time of psilocin C_{max} (i.e., at 2 hours post dose) in nearly all psilocybin dose groups, and mean ΔHR decreased with decreasing psilocin concentration at subsequent time points. Instances of bradycardia or tachycardia were unimodal, with no swing between bradycardia and tachycardia after a given dose. The episodes of bradycardia and tachycardia reported in recent studies at NYU, Johns Hopkins, and Wisconsin were asymptomatic (“mild” or CTCAE Grade 1) and did not require treatment (*Tryp IB, 2021 v2.1*).

Headache: Mild headaches are common within the 24 hours after a dose of psilocybin. No auras orphoto/phonophobia are associated with these headaches, which respond well to a single dose of acetaminophen. The headaches did not appear to be dose-related in one study, with no higher incidence after doses of 0.6 mg/kg versus 0.3 mg/kg.

2.3.2 KNOWN POTENTIAL BENEFITS

This patient population has few treatment options available to them. The medical risk induced with their disease can impact their quality of life and potentially their length of life due to their level of obesity. Based on other studies with psilocybin, the potential short-term benefit is to decrease their anxiety regarding food and their hyperphagia which can become the only focus of their lives. In the long term, management of this disease can allow the patient to make eating and other lifestyle changes which could result in significant weight loss and the health benefits that accompany that loss.

2.3.3 ASSESSMENT OF POTENTIAL RISKS AND BENEFITS

It is not believed that this patient population will have an increased risk with the study medication over any other population. The same pair of therapists will follow individual participants throughout their preparatory, dosing, and integration study experience.

3 OBJECTIVES AND ENDPOINTS

The primary objective of this study assessed by the following endpoints is to:

1. Assess the safety of a single dose of TRP-8802 in participants with BED during the TRP-8802 dosing session, and through 12 weeks following dosing (i.e., Week 14) (see Section 8.2).
   - i Nature and severity of AEs
   - ii Changes in vital signs (BP, heart rate); physical examination; ECG; clinical laboratory parameters (hematology, blood chemistry); C-SSRS

The secondary objectives of this study assessed by the following endpoints are to:

1. Evaluate the feasibility of inducing the psychedelic state with TRP-8802 in a BED population.
   - i Magnitude and duration of TRP-8802-induced dissociative effects in participants with BED using the MEQ30 and MRS (see Section 8.1).
2. Determine the preliminary clinical activity and the effects of TRP-8802 in conjunction with psychotherapy on the frequency of binge eating episodes and other weight-related indicators in a BED population through 4 weeks following dosing (i.e., Week 6) (see Section 8.1) in:
   - i Frequency of binge eating episodes as measured by a modified Eating Questionnaire (2 questions, daily)
   - ii CGI-I scale
   - iii Waist circumference
   - iv BMI

The exploratory objectives of this study assessed by the following endpoints are to:

1. Determine the effects of TRP-8802 on multiple ancillary indicators of clinical activity in a BED population through 12 weeks following dosing (i.e., Week 14) (see Section 8.1):
   - i Binge eating episodes (Eating Questionnaire), CGI-I, waist circumference, and BMI at 8 and 12 weeks following dosing (i.e., Weeks 10 and 14, respectively).
   - ii Patient-reported outcomes of Binge Eating Scale (BES), Patient Global Impression-Improvement (PGI-I), Hospital Anxiety and Depression Scale (HADS), and Acceptance and Action Questionnaire-II (AAQ-II) at 4, 8, and 12 weeks following dosing (i.e., Weeks 6, 10, and 14, respectively).
   - iii Neurophysiologic biomarkers: Resting state functional connectivity (fed and fasted), task-related functional activation and connectivity during a food cue

reactivity task (fed and fasted), voxel-based morphometry (grey matter volume/structure); resting electroencephalogram (EEG) at 4 weeks dosing (i.e., Week 6).

iv Metabolic biomarkers (plasma): leptin, adiponectin, ghrelin, insulin, and glucose; and HOMA-IR (Homeostatic Model Assessment of Insulin Resistance) at 4, 8, and 12 weeks following dosing (i.e., Weeks 6, 10, and 14, respectively).

2. Assess the relationship between clinical activity and the intensity of the psychedelic experience using the MEQ30, the CEQ, the EBI, and the MRS (see Section 8.1).

4 STUDY DESIGN

4.1 OVERALL DESIGN

This is a single center phase 2a open label study to assess the safety and feasibility of a single dose of TRP-8802 in subjects with BED. Subjects will undergo screening, preparation therapy sessions, dosing, integration therapy sessions and follow up for 12 weeks following the dose of TRP-8802. The total participation in the study will be up to approximately 5 months.

4.2 SCIENTIFIC RATIONALE FOR STUDY DESIGN

Binge eating disorder is a serious medical illness marked by severe disturbance to a person's control over their eating behaviors and high anxiety around food. BED is characterized by abnormal neural responses to food, especially highly palatable foods (*Boswell et al., 2021*; *Citrome 2019*; *Donnelly et al., 2018*). Additionally, individuals affected by BED suffer from severe anxiety and can have significant compulsive and impulsive behaviors, particularly around eating (*Samodien 2021*; *Guerdjikova et al., 2019*). Self-mutilatory behaviors, such as skin picking can be seen in BED, presumably associated with abnormal neuronal connectivity (Houazene 2021). Because psilocybin alters neuronal connections the potential opportunities of this therapy include treatment of overall anxiety, anxiety around food, compulsive and impulsive behaviors, self-mutilatory behaviors, and repetitive and intrusive thoughts, especially about food in people with BED (*Odland et al, 2021*; *Romeo et al, 2021*; *Johnson and Griffiths 2017*).

4.3 JUSTIFICATION FOR DOSE

A single, oral, escalating dose of psilocybin (0.3, 0.45, and 0.6 mg/kg) was used to evaluate the PK in 12 healthy adults (*Brown 2017*). The PK of psilocin were linear within the 2-fold range of doses, and the elimination half-life of psilocin was 3 h (SD 1.1). For this study, the single dose of 25 mg is a dose level that is not only supported by the Brown publication but also falls well within the dose range that has been extensively utilized in therapeutic trials in various indications (most recently, for example, *Carhart-Harris, 2021*). In previous studies, doses ranged from 19-59 mg based on mg/kg dosing (*Nicholas 2018*, *Brown, 2017*). All doses were well tolerated both physically and psychologically with no serious adverse events (SAEs) during or

within 30 days of the dose. For more detailed information regarding the extensive published clinical dosing-finding literature, refer to the current version of the TRP-8802 Clinical Investigator Brochure Section 5.3 and Table 4.

4.4 END OF STUDY DEFINITION

A participant is considered to have completed the study if he or she has completed all phases of the study including the last visit or the last scheduled procedure shown in the Schedule of Activities (SOA), Section 1.3.

The end of the study is defined as completion of the last visit or procedure shown in the SOA by the last subject.

5 STUDY POPULATION

5.1 INCLUSION CRITERIA

To participate in this study, subjects must meet all of the following criteria:

1. Meet DSM-5 criteria for BED.
2. Age ≥18 and ≤64 years.
3. Provision of signed and dated informed consent form.
4. Stated willingness to comply with all study procedures and availability for the duration of the study.
5. Medically stable in the judgment of the PI, as determined by screening medical, physical examination, ECG, and routine laboratory tests including blood and urinalysis.
6. For females of reproductive potential: use of highly effective contraception for at least 1 month prior to screening and agreement to use such a method during study participation and for an additional 4 weeks following the dose of TRP-8802. Adequate birth control methods include intrauterine device; injected, implanted, intravaginal, or transdermal hormonal method; oral hormones plus a barrier contraception; abstinence; vasectomized sole partner; or double barrier contraception.
7. For males of reproductive potential: use of condoms or other methods to ensure effective contraception with partner through 90 days post dose.
8. Agree to consume approximately the same amount of caffeine-containing beverage (e.g., coffee, tea) that he/she consumes on a usual morning, before arriving at the research unit on the morning of the drug session day. If the participant does not routinely consume caffeinated beverages, he/she must agree not to do so on the dosing session day.

9. Agree to refrain from using any psychoactive drugs, including alcoholic beverages for a minimum of 1 week prior to drug administration.
10. Agree that for one week before the drug session, including the morning of the session, he/she will refrain from taking any nonprescription medication, nutritional supplement, herbal supplement, or as needed (PRN) prescription medication except when approved by the study investigators. Exceptions will be evaluated by the study investigators and will include acetaminophen, non-steroidal anti-inflammatory drugs, and common doses of vitamins and minerals.

5.2 EXCLUSION CRITERIA

To participate in this study, subjects must not meet any of the following criteria:

1. Significant suicide risk as defined by either suicidal ideation as endorsed on items 4 or 5 on the C-SSRS within the past year, at Screening or at Baseline; or suicidal behaviors within the past year; clinical assessment of significant suicidal risk during subject interview.
2. Participation in another concurrent clinical study or within the preceding month.
3. Women who are pregnant or who intend to become pregnant during the study or who are currently nursing.
4. Vital signs, averaged over 3 readings within 15 minutes, of systolic BP >139 mm Hg, diastolic BP >89 mm Hg, or heart rate >90 bpm.
5. Have any of the following cardiovascular conditions: uncontrolled hypertension, coronary artery disease, congenital long QT syndrome, cardiac hypertrophy, cardiac ischemia, congestive heart failure, prior myocardial infarction, tachycardia, artificial heart valve, QTc >450 msec at screening, any other clinically significant screening ECG abnormality, or any other significant cardiovascular condition.
6. Presence of a gastrointestinal disease that could interfere with absorption of orally-administered TRP-8802.
7. Have epilepsy.
8. Meet DSM-5 criteria for schizophrenia spectrum or other psychotic disorders, including major depressive disorder with psychotic features, or Bipolar I or Bipolar II Disorder.
9. Family history of psychosis.
10. Meet DSM-5 criteria for a moderate or severe alcohol or drug use disorder.
11. Positive urine drug screen or alcohol breath test at screening. A repeat test can be conducted at screening or Day -1 at the discretion of the PI or delegate.

12. Prior adverse effects from psilocybin.
13. Currently taking or expected to need prior to the dosing session, UGT1A9 or 1A10 inhibitors (e.g., regorafenib, rifampicin, phenytoin, eltrombopag, mefenamic acid, diflunisal, niflumic acid, sorafenib, isavuconazole, deferasirox, ginseng), and aldehyde or alcohol dehydrogenase inhibitor (e.g., disulfiram).
14. Currently taking or testing positive on urine drug screen, drugs of abuse such as amphetamines, buprenorphine, benzodiazepines, cocaine, methamphetamines, Ecstasy (MDMA), morphine, methadone, oxycodone, marijuana, ethyl glucuronide, fentanyl, tramadol, and synthetic cannabinoids (K2).
15. Currently taking on a regular (e.g., daily) basis any medications having a primary centrally-acting serotonergic effect, including SSRIs, MAOIs, or serotonin-acting dietary supplements (such as 5-hydroxy tryptophan or St. John's wort). For individuals who have intermittent or PRN use of such medications, the dosing session will not be conducted until at least 5 half-lives of the agent have elapsed after the last dose.
16. fMRI subjects: Contraindications to fMRI procedures, per institutional policy.

5.3 SCREEN FAILURES

Screen failures are defined as participants who consent to participate in the clinical study but do not receive the study medication. A minimal set of screen failure information is required to ensure transparent reporting of screen failure participants, to meet the Consolidated Standards of Reporting Trials (CONSORT) publishing requirements and to respond to queries from regulatory authorities. Minimal information includes demography, screen failure details, eligibility criteria, and any SAE.

Individuals who do not meet the criteria for participation in this study (screen failure) because of a urine drug screen may be rescreened. Rescreened participants should be assigned the same participant number as for the initial screening.

5.4 STRATEGIES FOR RECRUITMENT AND RETENTION

This is a single center study and the PI will be inviting the current and new patients to participate in the study.

6 STUDY INTERVENTION

6.1 STUDY INTERVENTION(S) ADMINISTRATION

6.1.1 STUDY INTERVENTION DESCRIPTION

Psilocybin 3-[2-(dimethylamino) ethyl]-1H-indol-4-yl] dihydrogen phosphate is a natural product produced by numerous species of *Psilocybe* mushrooms. The phosphate group is enzymatically

cleaved in the body to produce psilocin, an agonist at a variety of serotonin receptors, the most important of which, for its behavioral effects, is the 5-HT2A receptor (*Carhart-Harris et al., 2014*; *Nichols, 2004*). Psilocybin was first isolated from *Psilocybe* mushrooms in 1957, followed by *de novo* synthesis in 1958 (*Passie et al., 2002*). It was marketed worldwide in the 1960s as *Indocybin™* for experimental and psychotherapeutic purposes. Although it was well tolerated and demonstrated potentially useful effects, it was classified as a controlled substance in the US, and placed in Schedule I in 1970, thus effectively removed from clinical use or scientific study. Psilocybin, and similar drugs such as lysergic acid diethylamide (LSD) and mescaline, fall into a pharmacological class that are referred to in this application as "classic psychedelics" to differentiate them from other psychoactive substances (ex. 3,4-methylenedioxy-methamphetamine; MDMA) that have different psychological/behavioral effects and different adverse effect profiles and risk/benefit ratios than psilocybin (*Carhart-Harris & Nutt, 2013*; *Nutt et al., 2010*). Psilocybin is currently only available for clinical investigation and is provided by Usona Institute in 25 mg capsules.

6.1.2 PSYCOHTHERAPY, DOSING, AND IMAGING

6.1.2.1 SET AND SETTING

Due to the psychoactive nature of psilocybin, the safety of participants in clinical studies can be enhanced by testing psilocybin within a "set and setting" protocol (*Lyons & Carhart-Harris, 2018*). By addressing the *set* (the emotional/cognitive/behavioral state/mindset and expectations of study participants just prior to psilocybin exposure) and *setting* (the physical environment in which the exposure occurs) of the experience, the risk of the subject reporting an event which was distressing or injuring themselves can be reduced. This approach generally incorporates three components: 1) preparation, 2) drug session, and 3) post session meetings to integrate the classic hallucinogen experience. Details of this procedure from pre-dose to post-dose integration is provided in Appendix A: Dosing and Therapy Sessions.

Prior to dose of TRP-8802, participants undergo pre-exposure preparation sessions designed to build rapport with the therapists who will be present during the drug exposure session and to identify personal themes and struggles that might be especially likely to impact the session experience. The drug session itself is conducted by two therapists (typically a male and a female) who are present throughout the session. Sessions are typically conducted in a room designed to be quiet, comfortable, and aesthetically pleasing, and participants are encouraged to wear eyeshades and listen to a program of music through headphones during the drug exposure to aid them in focusing their attention inward.

6.1.2.2 DOSING AND DOSING SESSION MONITORING

During the drug session, participants will arrive at the research unit in the morning. Participants will be instructed to consume a low-fat breakfast (e.g., 300-500 calories) at least 1 hour before reporting to the research unit. The session will be rescheduled if a friend or family member of

the participant is not available to accompany them home after being discharged from the research unit.

A single oral dose of 25 mg will be administered by trained clinical staff in the research unit. Oral TRP-8802 will be provided in 25 mg capsules.

The session monitors are trained to reassure the participants if they experience acute anxiety, agitation, paranoia, or panic. However, in the unlikely event that those symptoms do not respond to reassurance, or if a participant experiences an AE, one of the session monitors will contact the on-call study physician. Unless noted otherwise, the study physician will use their clinical judgment to determine whether to transfer the participant to the University emergency room for the most appropriate medical intervention.

Psychiatric emergency: An oral benzodiazepine (e.g., lorazepam) will be available at the University emergency room for treatment of panic or anxiety. An oral antipsychotic (e.g., risperidone) will be available for treatment of psychosis or severe agitation.

Hypertension: If a participant's BP is >200 mm Hg systolic or >110 mm Hg diastolic for more than 15 minutes (i.e., 4 consecutive readings) they will be transferred to the emergency room whether or not they are symptomatic. Labetalol or similar drug will be available at the University emergency room for treatment of hypertension.

Acute chest pain: If a participant develops new-onset chest pain, they will be evaluated in-person by the study physician to determine whether the pain is cardiovascular or musculoskeletal, or reflects acute panic/anxiety. Vital signs and ECG will be monitored (and compared to baseline measurements) for evidence of acute ischemia. Based on physician judgment, the participant may be transferred to the University emergency room for additional care.

Headache: Ibuprofen (400 mg PO) or similar will be available for treatment of headache.

If the study physician decides that the use of a rescue medication is warranted then a research pharmacist at the University emergency room will dispense the medication and the study physician or a member of the nursing staff will administer the medication to the participant.

Participants will not be allowed to leave the research unit until the study staff makes the judgment that the effects of the medication have completely subsided. The discharge criteria are as follows (all of the following must be true):

- At least 8 hours have elapsed since TRP-8802 dosing
- A responsible friend/family member is available to accompany the participant home
- The participant's BP and heart rate have returned to pre-drug levels
- The participant is deemed by study staff to be free of any acute drug effects

- The participant believes they have returned to their psychological baseline
- Study staff judge that it is safe to discharge the participant
- The participant expresses a readiness to go home

6.1.2.3 POST-DOSE PSYCHOTHERAPY

After the dosing session, participants are engaged in in-person integration sessions the day after the dosing. Another integration session will be held by video or in-person approximately 7 days after dosing. Further details on the post dose psychotherapy integration sessions are provided in Appendix A: Dosing and Therapy Sessions.

All therapists involved in this process will be trained by an independent group, and the Psychotherapy Manual for all stages of the process will be provided for dealing with patients administered a psychedelic drug.

6.1.2.4 FUNCTIONAL MAGNETIC RESONANCE IMAGING

Functional magnetic resonance imaging will be performed on participants for whom the procedure is not contraindicated by institutional policy.

Participants will arrive at the University of Florida Advanced Magnetic Resonance Imaging and Spectroscopy facility after fasting (except for water) since midnight. Participants will be positioned head-first in the supine position in the MRI scanner with a 64-channel head coil. They will rate their current level of hunger using a 100-point visual analog scale (VAS) anchored from “Not at all hungry” to “Hungriest imaginable”. First, a blood oxygen level-dependent (BOLD) resting state functional scan (~9 minutes) will be acquired. Participants will be instructed to stay as still as possible, let their thoughts wander, keep their eyes fixated on a central cross displayed on a display, and do their best to stay awake. Next, participants will complete a food cue reactivity task during BOLD functional imaging acquisition. During this task, participants will view images of highly-processed foods (e.g., pizza), minimally-processed foods (e.g., apple), and neutrally valent pictures of household objects (e.g., light bulb) and will be instructed to think about how much they desired each item. This task will require approximately 14 minutes. After task completion, participants will be withdrawn from the MRI bore and provided with a small meal (i.e., meal replacement bar and no more than 12 ounces of water [fed state]). Once complete, they will be repositioned in the MRI and the hunger VAS, resting-state scan and cue reactivity task will be repeated. The order of the resting state scans and cue reactivity task will be counterbalanced. Finally, we will collect a high-resolution T1-weighted structural brain image (~4 minutes). In total, MRI testing will require approximately 1 hour.

6.1.2.5 ELECTROENCEPHALOGRAM

Resting EEG will be acquired in an exam room at the University of Florida Advanced Magnetic Resonance Imaging and Spectroscopy facility. Following electrode cap application, the site will acquire 10 minutes each of eyes open and eyes closed resting EEG in counterbalanced order.

6.2 PREPARATION/HANDLING/STORAGE/ACCOUNTABILITY

6.2.1 ACQUISITION AND ACCOUNTABILITY

Medication will be provided to the investigator and must be maintained in a controlled and locked location until a subject dosing session. These should be stored consistent with institutions requirements for controlled substances.

6.2.2 FORMULATION, APPEARANCE, PACKAGING, AND LABELING

TRP-8802 is manufactured by Usona Institute and provided as a 25 mg capsules (size 2, hydroxypropyl methyl cellulose (HPMC), white). TRP-8802 capsules are packaged individually into high-density polyethylene bottles (30 cc).

6.2.3 PRODUCT STORAGE AND STABILITY

TRP-8802 bottles must be maintained at room temperature in a locked, secure location within the research pharmacy at the site and in accordance with Drug Enforcement Agency (DEA) regulations. Study staff with access to the TRP-8802 inventory will be pre-defined.

6.3 MEASURES TO MINIMIZE BIAS: RANDOMIZATION AND BLINDING

This study is an open label study and all subjects will be aware that they are taking the study medication. There may be some bias associated with subject selection to participate in the study.

6.4 STUDY INTERVENTION COMPLIANCE

This study is a single dose study under the observation of a clinician or therapists and therefore it will be clear whether the study medication is taken.

6.5 CONCOMITANT THERAPY

For this protocol, a prescription medication is defined as a medication that can be prescribed only by a properly authorized/licensed clinician. Medications to be reported in the Case Report Form (CRF) are concomitant prescription medications, over-the-counter medications and supplements.

7 STUDY INTERVENTION DISCONTINUATION AND PARTICIPANT DISCONTINUATION/WITHDRAWAL

7.1 DISCONTINUATION OF STUDY INTERVENTION

Discontinuation from the study process prior to last integration session does not mean discontinuation from the study, and remaining study procedures should be completed as indicated by the study protocol. If a clinically significant finding is identified (including, but not limited to changes from baseline) after enrollment, the investigator or qualified designee will determine if any change in participant management is needed. Any new clinically relevant finding will be reported as an AE.

The data to be collected at the time of study intervention discontinuation will include all assessments associated with early discharge on the SOA.

7.2 PARTICIPANT DISCONTINUATION/WITHDRAWAL FROM THE STUDY

Participants are free to withdraw from participation in the study at any time upon request. An investigator may discontinue or withdraw a participant from the study for any reason, including protection of patient safety, judgment that it would be in the subject's best interests, or a participant's lack of compliance with protocol requirements.

The reason for participant discontinuation or withdrawal from the study will be recorded on the CRF. Subjects who sign the informed consent form but do not receive the study intervention may be replaced. Subjects who sign the informed consent form, and receive the study intervention, and subsequently withdraw, or are withdrawn or discontinued from the study, may be replaced.

7.3 LOST TO FOLLOW-UP

A participant will be considered lost to follow-up if he or she fails to return for any scheduled visits and is unable to be contacted by the study site staff.

The following actions must be taken if a participant fails to return to the clinic for a required study visit:

- The site will attempt to contact the participant and reschedule the missed visit within three days for follow up visits and counsel the participant on the importance of maintaining the assigned visit schedule and ascertain if the participant wishes to and/or should continue in the study.
- Before a participant is deemed lost to follow-up, the investigator or designee will make every effort to regain contact with the participant (where possible, 3 telephone calls and, if necessary, a certified letter to the participant's last known mailing address or local equivalent methods). These contact attempts should be documented in the participant's medical record or study file.
- Should the participant continue to be unreachable, he or she will be considered to have withdrawn from the study with a primary reason of lost to follow-up.

8 STUDY ASSESSMENTS AND PROCEDURES

8.1 CLINICAL ACTIVITY ASSESSMENTS

Planned timing for all assessments is provided in the SOA. Where appropriate, visit windows of +/- 2 days are allowed.

- Frequency of Binge Eating (daily): Frequency of binge eating will be assessed using 2 items from the Eating Questionnaire (*Fairburn & Beglin, 1994*). For 4 weeks before and 4 weeks after TRP-8802 dosing, subjects will be asked to record the number of binge eating episodes daily, once a day, using 2 items:

  i Over the past 24 hours, how many times have you eaten what other people would regard as an unusually large amount of food (given the circumstances)?

  ii On how many of these times did you have a sense of having lost control over your eating (at the time that you were eating)?

- Frequency of Binge Eating (every 4 weeks): Frequency of binge eating will be assessed using 3 items from the Eating Questionnaire (*Fairburn & Beglin, 1994*) to assess durability. At Screening, Week 10, and Week 14, subjects will be asked to record the number of binge eating episodes over the previous 4 weeks using the standard 3 questions:

  i Over the past 28 days, how many times have you eaten what other people would regard as an unusually large amount of food (given the circumstances)?

  ii On how many of these times did you have a sense of having lost control over your eating (at the time that you were eating)?

  iii Over the past 28 days, on how many DAYS have such episodes of overeating occurred (i.e., you have eaten an unusually large amount of food and have had a sense of loss of control at the time)?

- Clinician Global Impression – Improvement (CGI-I) (*Guy, 1976*) is a 7 point scale that requires the clinician to assess how much the subject's illness has improved or worsened relative to the baseline state using a 7-point scale from very much improved (1) to very much worse (7).

- Waist circumference will be assessed.

- Body mass index will be calculated based on subject’s height and weight.

- Binge Eating Scale (BES) (*Gormally et al., 1982*) is a 16 item questionnaire assessing the presence of certain binge eating behaviors which may be indicative of an eating disorder.

- Patient Global Impression – Improvement (PGI-I) (*Guy, 1976*) is 7 point scale that is a single question asking the patient to rate their condition now, as compared with how it was prior to before beginning treatment from very much improved (1) to very much worse (7).

- Hospital Anxiety Depression Scale (HADS) (*Zigmond & Snaith, 1983*) is a 14 item patient rating scale for detecting states of depression and anxiety in the setting of a hospital medical outpatient clinic. Seven items are specific to each and can be evaluated as subscales.

- Emotional Breakthrough Inventory (EBI) (*Roseman et al., 2019*): The six item inventory of emotional breakthrough is validated as an important component of the acute psychedelic experience that appears to be a key mediator of subsequent longer-term psychological changes. When the EBI is combined with the MEQ30 and CEQ, they significantly predicted subsequent changes in well-being.

- Acceptance and Action Questionnaire-II (AAQ-II) (*Bond et al., 2011*): Assesses the construct referred to as, variously, acceptance, experiential avoidance, and psychological inflexibility.

- EEG**:** Explosive synchronization of network activity at rest, Power spectrum analysis (frequency bands).

- fMRI: Resting state functional connectivity (fed and fasted), task-related functional activation and connectivity during a food cue reactivity task (fed and fasted), voxel-based morphometry (grey matter volume/structure).

- Metabolic biomarkers**:** Blood samples (26 mL of blood at each of 4 visits [104 mL total]) will be collected to evaluate metabolic biomarkers from plasma, including: leptin, adiponectin, ghrelin, insulin, glucose and HOMA-IR (Homeostatic Model Assessment of Insulin Resistance).

- Mystical Experience Questionnaire (MEQ30) (*Barrett et al., 2015*): This is a 30-item questionnaire to survey mystical-type experiences occasioned by TRP-8802. Scores on the MEQ30 positively predict persisting change in attitudes, behavior and well-being attributed to experiences with psilocybin while controlling for the contribution of the participant-rated intensity of the drug effects.

- Challenging Experiences Questionnaire (CEQ) (*Barrett et al., 2016)*: Scale to assess the meaningfulness and difficulty of their reported TRP-8802 experience, the degree to which the experience was spiritually significant to them, the effect of the challenging experience on their well-being or life satisfaction and whether they had struggled with a psychiatric disorder at some point before their reported challenging experience.

- Monitor Rating Scale (MRS) (*Griffiths et al., 2006*): Questionnaire completed by session monitors and involves rating and scoring dimensions of participant's behavior or mood during dosing session.

8.2 SAFETY ASSESSMENTS

Safety assessments will be conducted to monitor AEs, including SAEs, protocol specific safety laboratory assessments, protocol specific vital signs and other tests/questionnaires that are deemed crucial to the safety evaluation of the study. Planned timing for all safety assessments is provided in the SOA.

- Physical examination: height and weight, organ system
- Vital signs: heart rate, BP
- Blood chemistry (requiring 7 mL of blood at each of 7 visits [49 mL total]): Na+, K+, Cl-, HCO3-, Ca++, Mg++, P, BUN, creatinine, glucose, total bilirubin, albumin, ALT, AST, GGT, CK, LDH, alkaline phosphatase
- Hematology (requiring 3 mL of blood at each of 7 visits [21 mL total]): CBC with white cell differential and platelet count
- Urinalysis: include pH, specific gravity, protein, occult blood, glucose, and ketones plus microscopic examination of sediment for RBC, WBC, epithelial cells, casts, crystals, and bacteria
- ECG: 12-Lead (in triplicate at all time points tested)
- C-SSRS (*Posner et al., 2011*): The Columbia-Suicide Severity Rating Scale (C-SSRS) is a questionnaire used for suicide assessment designed to distinguish the domains of suicidal ideation and suicidal behavior through four constructs, severity of ideation, intensity of ideation, behavior subscale, and the lethality subscale.
- AEs

8.3 ADVERSE EVENTS AND SERIOUS ADVERSE EVENTS

8.3.1 DEFINITION OF ADVERSE EVENTS (AE)

Adverse event means any untoward medical occurrence associated with the use of an intervention in humans, whether or not considered intervention-related (21 CFR 312.32 (a)).

An AE can be any unfavorable and unintended sign (e.g., an abnormal laboratory finding), symptom, or disease temporarily associated with the use of a drug and does not imply any judgment about causality.

8.3.2 DEFINITION OF SERIOUS ADVERSE EVENTS (SAE)

An AE or suspected adverse reaction is considered "serious" if, in the view of either the investigator or sponsor, it results in any of the following outcomes:

- Death
- a life-threatening AE
- inpatient hospitalization or prolongation of existing hospitalization
- a persistent or significant incapacity or substantial disruption of the ability to conduct normal life functions,
- or a congenital anomaly/birth defect
- Important medical events that may not result in death, be life-threatening, or require hospitalization may be considered serious when, based upon appropriate medical judgment, they may jeopardize the participant and may require medical or surgical intervention to prevent one of the outcomes listed in this definition. Examples of such medical events include allergic bronchospasm requiring intensive treatment in an emergency room or at home, blood dyscrasias or convulsions that do not result in inpatient hospitalization, or the development of drug dependency or drug abuse.

8.3.3 CLASSIFICATION OF AN ADVERSE EVENT

8.3.3.1 SEVERITY OF EVENT

For each AE, the PI should make an assessment regarding the severity of event:

- **Mild** – Events require minimal or no treatment and do not interfere with the participant's daily activities.
- **Moderate** – Events result in a low level of inconvenience or concern with the therapeutic measures. Moderate events may cause some interference with functioning.
- **Severe** – Events interrupt a participant's usual daily activity and may require systemic drug therapy or other treatment. Severe events are usually potentially life-threatening or incapacitating. Of note, the term "severe" does not necessarily equate to "serious".

8.3.3.2 RELATIONSHIP TO STUDY INTERVENTION

For each AE, the PI should make an assessment regarding the relationship to the medication. The participant should be examined and the PI should evaluate the participant based on temporal relationship and his/her clinical judgment. The degree of certainty about causality will be graded using the categories below. In a clinical study, the study product must always be suspect.

- **Definitely Related** – There is clear evidence to suggest a causal relationship, and other possible contributing factors can be ruled out. The clinical event, including an abnormal

laboratory test result, occurs in a plausible time relationship to study intervention administration and cannot be explained by concurrent disease or other drugs or chemicals. The response to withdrawal of the study intervention (dechallenge) should be clinically plausible. The event must be pharmacologically or phenomenologically definitive, with use of a satisfactory rechallenge procedure if necessary.

- **Probably Related** – There is evidence to suggest a causal relationship, and the influence of other factors is unlikely. The clinical event, including an abnormal laboratory test result, occurs within a reasonable time after administration of the study intervention, is unlikely to be attributed to concurrent disease or other drugs or chemicals, and follows a clinically reasonable response on withdrawal (dechallenge). Rechallenge information is not required to fulfill this definition.
- **Possibly Related** – There is some evidence to suggest a causal relationship (e.g., the event occurred within a reasonable time after administration of the trial medication). However, other factors may have contributed to the event (e.g., the participant's clinical condition, other concomitant events). Although an AE may rate only as "possibly related" soon after discovery, it can be flagged as requiring more information and later be upgraded to "probably related" or "definitely related", as appropriate.
- **Unlikely related** – A clinical event, including an abnormal laboratory test result, whose temporal relationship to study intervention administration makes a causal relationship improbable (e.g., the event did not occur within a reasonable time after administration of the study intervention) and in which other drugs or chemicals or underlying disease provides plausible explanations (e.g., the participant's clinical condition, other concomitant treatments).
- **Not Related** – The AE is completely independent of study intervention administration, and/or evidence exists that the event is definitely related to another etiology. There must be an alternative, definitive etiology documented by the clinician.

If there is any doubt as to whether a clinical observation is an AE, the event should be reported.

8.3.3.3 EXPECTEDNESS

An AE or suspected adverse reaction is considered "unexpected" if it is not listed in the IB or is not listed at the specificity or severity that has been observed.

The PI will be responsible for determining whether an AE is expected or unexpected. An AE will be considered unexpected if the nature, severity, or frequency of the event is not consistent with the risk information previously described for the study intervention.

8.3.3.4 ADVERSE EVENTS RELATED TO DRUGS WITH POTENTIAL FOR ABUSE

Possible cases of abuse (e.g., participants taking the drug for non-therapeutic purposes for psychoactive effects such as high or euphoria) will be reported as AEs. The unauthorized use of the TRP-8802 by individuals other than the participants (e.g., by study staff) will also be reported as AEs. If such AEs should occur, data for all reports of abuse, overuse, lost/stolen/missing, or unaccounted for TRP-8802 will be reported in tabular form. Details regarding investigator training to capture cases of abuse, misuse, and addiction are provided in the Psychotherapy Manual.

8.3.4 TIME PERIOD AND FREQUENCY FOR EVENT ASSESSMENT AND FOLLOW-UP

All AEs including local and systemic reactions not meeting the criteria for SAEs will be captured on the appropriate CRF. Information to be collected includes event description, time of onset, clinician's assessment of severity, relationship to study product (assessed only by those with the training and authority to make a diagnosis), and time of resolution/stabilization of the event. All AEs occurring while on study must be documented appropriately regardless of relationship. All AEs will be followed to adequate resolution.

Any medical condition that is present at the time that the participant is screened will be considered as baseline and not reported as an AE. However, if the study participant's condition deteriorates at any time during the study, the worsening of the condition will be recorded as an AE.

Changes in the severity of an AE will be documented to allow an assessment of the duration of the event at each level of severity to be performed. AEs characterized as intermittent require documentation of onset and duration of each episode.

An event is to be collected if the start date occurs any time after informed consent is obtained until 7 (for non-SAEs) or 30 days (for SAEs) after the last day of study participation. At each study visit, the investigator will inquire about the occurrence of AE/SAEs since the last visit. Events will be followed for outcome information until resolution or stabilization.

8.3.5 ADVERSE EVENT REPORTING

Adverse Events which meet the criteria for SAEs will be reported within 24 hours of site awareness. Specific instructions and forms will be provided to the site prior to first subject enrollment.

8.3.6 SERIOUS ADVERSE EVENT REPORTING

All SAEs must be reported to the sponsor or designee within 24 hours of awareness, whether or not considered study intervention related, including those listed in the protocol or investigator brochure and must include an assessment of whether there is a reasonable possibility that the study intervention caused the event. Study endpoints that are SAEs (e.g., all-cause mortality) must be reported in accordance with the protocol unless there is evidence suggesting a causal

relationship between the study intervention and the event (e.g., death from anaphylaxis). In that case, the investigator must immediately report the event to the sponsor.

All SAEs will be followed until satisfactory resolution or until the site investigator deems the event to be chronic or the participant is stable. AE/SAE supporting documentation of the event may be requested study sponsor and should be provided as soon as possible.

The study sponsor will be responsible for notifying the Food and Drug Administration (FDA) of any unexpected fatal or life-threatening suspected adverse reaction as soon as possible, but in no case later than 7 calendar days after the sponsor's initial receipt of the information. In addition, the sponsor must notify FDA and all participating investigators in an Investigational New Drug (IND) safety report of potential serious risks, from clinical studies or any other source, as soon as possible, but in no case later than 15 calendar days after the sponsor determines that the information qualifies for reporting.

8.3.7 REPORTING EVENTS TO PARTICIPANTS

Tryp Therapeutics or its designee will distribute IND Safety Reports to the investigator(s). Investigator(s) must submit safety reports as required by their Institutional Review Board (IRB)/IEC within timelines set by applicable regulations. Documentation of the submission to and receipt by the IRB/IEC IND Safety Reports must be retained in the site files.

8.3.8 REPORTING OF PREGNANCY

If a subject becomes pregnant during study participation, basic information about the pregnancy will be reported by the same method as an SAE. Pregnancy is itself not regarded as an AE unless there is a suspicion that the study medication may have interfered with the effectiveness of a contraceptive medication. If there are complications during the pregnancy, the complications are recorded as AEs. The subject will be asked to report the outcome of the pregnancy even if her study participation is discontinued, and the site should submit the information within 30 days after the outcome of the pregnancy. All reports of congenital abnormalities/birth defects are SAEs to be entered on the mother’s (ie subject’s SAE form). Spontaneous miscarriages should also be reported and handled as SAEs. Elective abortions without complications should not be reported as AEs. Partner pregnancies will not be reported.

9 STATISTICAL CONSIDERATIONS

9.1 STATISTICAL HYPOTHESES

This study documents the safety and the clinical activity of TRP-8802 in conjunction with psychotherapy in subjects with BED. There are no hypotheses associated with the primary endpoint of safety; the results of study are descriptive only.

9.2 SAMPLE SIZE DETERMINATION

The study is intended to gather preliminary clinical activity and feasibility of TRP-8802 treatment in conjunction with psychotherapy in adults with BED. The sample size is not selected based on power requirements for a formal hypothesis test. Accordingly, a sample of 10 participants is selected based on logistical and clinical considerations.

9.3 POPULATIONS FOR ANALYSES

The following analysis populations have been defined for this study: Safety Population, Full Analysis Set Population (FAS), and Per Protocol (PP) Population.

The Safety Population: The Safety Population consists of all participants for whom the experimental procedure is initialized under the study protocol. Participants who do not receive any amount of the study drug will still contribute to the safety analysis if the procedure was initialized but not completed, since all AEs of administration must be assessed. All safety analyses will utilize the Safety Population.

Full Analysis Set Population: The FAS Population will consist of all subjects who receive the single dose of study drug, and who have any post-baseline clinical assessments. The primary and secondary endpoint analyses will be performed using data from the FAS Population.

Per Protocol Population: The PP Population consists of the subset of the FAS Population who complete 4 weeks of follow up post dosing with no major protocol deviations (including but not limited to: failure to satisfy all inclusion and exclusion criteria, failure to adhere to all protocol-required restrictions and prohibitions, receipt of prohibited concomitant procedures or therapies, and non-compliance to protocol-specific procedures) as determined by a review of participant data. The analysis of clinical activity performed using data from the PP Population will be considered supportive of the FAS analysis.

9.4 STATISTICAL ANALYSES

9.4.1 GENERAL APPROACH

All descriptive statistical analyses will be performed using SAS (Version 9.4 or higher), unless otherwise noted. For categorical variables, the number and percentage within each category of the parameter will be calculated. For continuous variables, the number of subjects with no-missing data (n), mean, median, standard deviation (SD), minimum, and maximum values will be presented.

For the purpose of statistical analyses, a month is considered equivalent to 30 days, and a year is considered equivalent to 365 days.

9.4.2 ANALYSIS OF THE PRIMARY ENDPOINT(S)

In summary, vital signs (BP, heart rate) will be summarized at the dosing session, the day following dosing, and at Weeks 6, 10, and 14. Physical examination will be reported at Screening, the day following dosing, and at Weeks 6, 10, and 14. Furthermore, ECG, blood

chemistry, and hematology will be tabulated at scheduled visits. The results of C-SSRS questionnaires will be shown at the scheduled visits. Finally, the results of AEs will be presented.

9.4.3 ANALYSIS OF THE SECONDARY ENDPOINT(S)

The analysis of secondary endpoints will be conducted at follow up visits. The results of MRS and MEQ30 questionnaires will be shown at the scheduled visits. The reduction in the frequency of binge eating episodes measured daily will be tabulated through 4 weeks following dosing (Week 6). In addition, changes in both BMI and waist circumference, and the Physician-rated CGI-I scale through 4 weeks following dosing will be presented.

Furthermore, exploratory analyses will include CFB at all time points tested for the parameters below:

- Frequency of binge eating episodes as recalled over a 4-week period
- BES
- PGI-I
- CGI-I
- HADS
- EBI
- AAQ-II
- EEG (4 weeks post dosing):
    a. Explosive synchronization of network activity at rest
    b. Power spectrum analysis (frequency bands)
- fMRI (4 weeks post dosing):
    a. Functional connectivity (resting state; fed and fasted)
    b. Functional activation (food cue reactivity task; fed and fasted)
    c. Functional connectivity (food cue reactivity task; fed and fasted)
    d. Voxel Based morphometry (grey matter volume/structure)
- Metabolic biomarkers (plasma):
    a. leptin
    b. adiponectin

c. ghrelin

d. insulin

e. glucose

f. HOMA-IR (Homeostatic Model Assessment of Insulin Resistance)

An additional exploratory endpoint is the relationship between clinical activity and the intensity of the psychedelic experience as measured by MEQ30, CEQ, EBI, and MRS.

Again, continuous data will be summarized by the number of subjects with no-missing data (n), mean, median, SD, minimum, and maximum values. For categorical variables, the number and percent of each category will be displayed.

Biomarkers will be analyzed by using the appropriate statistical methodology for the given endpoint type and objective.

9.4.4 SAFETY ANALYSES

A treatment-emergent adverse event (TEAE) is any AE either reported for the first time or worsening of a pre-existing event after the dose of study drug. Analysis of AEs will be limited to TEAEs, but data listings will include all AEs regardless of their timing to study drug administration.

Adverse events will be coded into body systems and preferred terms using the Medical Dictionary for Regulatory Activities (MedDRA). Adverse events will be tabulated by the MedDRA preferred term and system organ class. All TEAEs and SAEs related to study treatment will be summarized and tabulated by preferred term, both overall and by severity, and by time period of onset. For tabulations by severity, only a subject’s most severe event within the category (e.g., overall, body system, or preferred term) will be counted. Adverse events will be categorized into those occurring within the following time periods (all time periods are inclusive, from the start of dosing, and the subject’s first occurrence within the time period will be included in the tabulations): 6 weeks (±2 days), 10 weeks (±2 days), 14 weeks (±2 days), and will include AEs collected at unscheduled visits.

9.4.5 BASELINE DESCRIPTIVE STATISTICS

The number and percentage of subjects screened, screen failure, subjects in each analysis population, completers, early withdrawal and reason for withdrawal by categories will be tabulated. Demographic variables, baseline characteristics, medical and medication history, and psychiatric history will be summarized either by descriptive statistics in terms of counts and percentages.

9.4.6 PLANNED INTERIM ANALYSES

There is no planned interim analysis for this study.

9.4.7 SUB-GROUP ANALYSES

There are no planned sub-group analyses for this study.

9.4.8 TABULATION OF INDIVIDUAL PARTICIPANT DATA

Subject listings of demographic and primary endpoint will be generated.

9.4.9 EXPLORATORY ANALYSES

Exploratory analyses will be conducted as described in Section 9.4.2.

10 SUPPORTING DOCUMENTATION AND OPERATIONAL CONSIDERATIONS

10.1 REGULATORY, ETHICAL, AND STUDY OVERSIGHT CONSIDERATIONS

10.1.1 INFORMED CONSENT PROCESS

10.1.1.1 CONSENT/ASSENT AND OTHER INFORMATIONAL DOCUMENTS PROVIDED TO PARTICIPANTS

IRB approved consent forms describing in detail the study intervention, study procedures, and risks are given to the participant and written documentation of informed consent is required prior to starting intervention/administering study intervention.

10.1.1.2 CONSENT PROCEDURES AND DOCUMENTATION

Informed consent is a process that is initiated prior to the individual's agreeing to participate in the study and continues throughout the individual's study participation. Consent forms will be IRB-approved and the participant will be asked to read and review the document. The investigator will explain the research study to the participant and answer any questions that may arise. A verbal explanation will be provided in terms suited to the participant's comprehension of the purposes, procedures, and potential risks of the study and of their rights as research participants. Participants will have the opportunity to carefully review the written consent form and ask questions prior to signing. The participants should have the opportunity to discuss the study with their family or surrogates or think about it prior to agreeing to participate. The participant will sign the informed consent document prior to any procedures being done specifically for the study. Participants must be informed that participation is voluntary and that they may withdraw from the study at any time, without prejudice. A copy of the informed consent document will be given to the participants for their records. The informed consent process will be conducted and documented in the source document (including the date), and the form signed, before the participant undergoes any study-specific procedures. The rights and welfare of the participants will be protected by emphasizing to them that the quality of their medical care will not be adversely affected if they decline to participate in this study.

10.1.2 STUDY DISCONTINUATION AND CLOSURE

This study may be temporarily suspended or prematurely terminated if there is sufficient reasonable cause. Written notification, documenting the reason for study suspension or termination, will be provided by the suspending or terminating party to study participants, investigator, the IND sponsor and regulatory authorities. If the study is prematurely terminated or suspended, the PI will promptly inform study participants, the IRB, and sponsor and will provide the reason(s) for the termination or suspension. Study participants will be contacted, as applicable, and be informed of changes to study visit schedule.

Circumstances that may warrant termination or suspension include, but are not limited to:

- Determination of unexpected, significant, or unacceptable risk to participants
- Demonstration of clinical activity that would warrant stopping
- Insufficient compliance to protocol requirements
- Data that are not sufficiently complete and/or evaluable
- Determination that the primary endpoint has been met
- Determination of futility

Study may resume once concerns about safety, protocol compliance, and data quality are addressed, and satisfy the sponsor, IRB and/or FDA.

10.1.3 CONFIDENTIALITY AND PRIVACY

Participant confidentiality and privacy is strictly held in trust by the participating investigators, their staff, and the sponsor(s) and their interventions. This confidentiality is extended to cover testing of biological samples and genetic tests in addition to the clinical information relating to participants. Therefore, the study protocol, documentation, data, and all other information generated will be held in strict confidence. No information concerning the study or the data will be released to any unauthorized third party without prior written approval of the sponsor.

All research activities will be conducted in as private a setting as possible.

The study monitor, other authorized representatives of the sponsor, representatives of the IRB, or regulatory agencies may inspect all documents and records required to be maintained by the investigator, including but not limited to, medical records (office, clinic, or hospital) and pharmacy records for the participants in this study. The clinical study site will permit access to such records.

The study participant’s contact information will be securely stored at each clinical site for internal use during the study. At the end of the study, all records will continue to be kept in a secure location for as long a period as dictated by the reviewing IRB, Institutional policies, or sponsor requirements.

Study participant research data, which is for purposes of statistical analysis and scientific reporting, will be transmitted to and stored by the sponsor or designee. This will not include the participant's contact or identifying information. Rather, individual participants and their research data will be identified by a unique study identification number. The study data entry and study management systems used by clinical sites and sponsor will be secured and password protected. At the end of the study, all study databases will be de-identified and archived by the sponsor.

10.1.4 KEY ROLES AND STUDY GOVERNANCE

**Principal Investigator**	**Medical Monitor**
Jennifer Miller, MD	Michael H. Silverman, MD
University of Florida	Tryp Therapeutics, Inc.
Gainesville, Florida 32608	Marblehead, MA 01945-2581
352-265-7337	781-631-8596
millejl@peds.ufl.edu	msilverman@biostrategics.com

10.1.5 SAFETY OVERSIGHT

Safety Review Committee

The SRC will be responsible for the assessment of safety and to make decisions with regards to study progression. The SRC will be composed of at least the PI, the study's Principal Psychologist, one medically qualified Sponsor representative (SRC Chair), and a biomedical professional with relevant experience and expertise. The SRC is responsible for reviewing study procedures, enrollment, drop-outs, and safety data such as AEs, vital signs, laboratory tests, ECGs, etc. The SRC members can ask questions and make comments and/or recommendations to the Sponsor.

The SRC will convene, at a minimum, to review safety data within 2 weeks of dosing the 1st, 2nd, 4th, 6th, and 8th subject to review overall safety information. More frequent meetings can be requested by the PI. The SRC may recommend to the Sponsor any of the following, as it seems appropriate:

1. Continuation of the study as planned.
2. Continuation of the study with modifications of procedures, such as dose, safety monitoring, or others.
3. Suspension of enrollment while further evaluating safety events; this is mandatory for any event meeting a Stopping Rule (see below).
4. Termination of the study.

AE Evaluation

- AE grading will be performed using FDA Guidance For Industry: Toxicity Grading Scale for Healthy Adult and Adolescent Volunteers Enrolled in Preventive Vaccine Clinical Trials
- During the dosing session, manifestations of the psychedelic experience will not be recorded as AEs, unless judged by the monitors to exceed the intensity and/or duration of expected reactions.

Stopping Rules

The stopping rules for safety are based on the posterior probability that the events listed below are too high:

- One SAE considered at least possibly related to the study drug.
- One Grade 3 or Grade 4 AE considered at least possibly related to the study drug.
- HPPD
- One QTcF prolongation, defined as QTcF ≥60 msec or prolonged QTcF >500 msec on ECG (average of triplicate tracings), regardless of the occurrence length of time, or an episode of *torsade de pointes*.

Trial dosing will be paused for a safety evaluation if the specific study drug-related AEs occur at a rate that convincingly exceeds 30% (applicable AEs that trigger this rule appear in the bullet list above). This stopping rule will halt dosing if the posterior probability of the event rate, which exceeds 30%, is 70% or higher. The prior probability for this safety monitoring rule is Beta distribution with parameter of 1 and 5, assuming one event out of 6 participants treated.

The estimated probability that an event rate exceeds 30% comes from combining this prior distribution with the observed number of participants experiencing an event to compute the posterior probability distribution of the event rate. The following table shows the resulting stopping rules for safety.

**Dosing will be paused if there are:**	3 Events	4 Events	5 Events
**and participants enrolled are between:**	3 to 4	5 to 7	8 to 10

For example, the stopping rule will call for pausing dosing if there are 3 events out of the first 3 to 4 dosed participants.

If any of the stopping rules in the table above is met, dosing will pause until the SRC reviews details of the event and/or additional data. If, after further review of the data, the SRC determines that no stopping rule has been met (e.g., AE determined to be unrelated to the study drug), then dosing may resume.

10.1.6 CLINICAL MONITORING

Clinical site monitoring is conducted to ensure that the rights and well-being of study participants are protected, that the reported study data are accurate, complete, and verifiable, and that the conduct of the study is in compliance with the currently approved protocol/amendment(s), with International Conference on Harmonisation Good Clinical Practice (ICH GCP), and with applicable regulatory requirement(s).

- Monitoring for this study will be performed by the sponsor, or designee.
- A centralized risk-based monitoring strategy will be deployed, with onsite being the primary means for monitoring for the TRYP-001 study. The monitoring performed, as detailed in the Clinical Monitoring Plan, will encompass the preliminary visits for assessing and initiating the sites, all subsequent interim monitoring visits, and site closures upon study completion. The aforementioned interim visits will be utilized through the life of the study to evaluate compliance with site data collection requirements as it pertains to the study's primary objective, secondary objectives, and study endpoints. Remote monitoring visits may also be used in addition or in place of any monitoring visit depending on study need and circumstance.
- For each visit conducted, a monitoring visit report will be provided to sponsor detailing the findings of said visit.
- Further details of the clinical site monitoring are documented in a Clinical Monitoring Plan (CMP). The CMP describes in detail who will conduct the monitoring, at what frequency monitoring will be done, at what level of detail monitoring will be performed, and the distribution of monitoring reports.
- Independent audits can be conducted by a vendor of the sponsor's choosing to ensure monitoring practices are performed consistently across all participating sites and that monitors are following the CMP.

10.1.7 QUALITY ASSURANCE AND QUALITY CONTROL

The clinical site is responsible for the quality management of study conduct, data and biological specimen collection, documentation and completion.

Quality control procedures will be implemented beginning with the data entry system and data QC checks that will be run on the database will be generated. Any missing data or data anomalies will be communicated to the site(s) for clarification/resolution.

Following written Standard Operating Procedures (SOPs), the monitors will verify that the clinical study is conducted and data are generated and biological specimens are collected, documented (recorded), and reported in compliance with the protocol, ICH GCP, and applicable regulatory requirements (e.g., Good Laboratory Practices [GLP]).

The investigational site will provide direct access to all study related sites, source data/documents, and reports for the purpose of monitoring and auditing by the sponsor, and inspection by local and regulatory authorities.

10.1.8 DATA HANDLING AND RECORD KEEPING

10.1.8.1 DATA COLLECTION AND MANAGEMENT RESPONSIBILITIES

Data collection is the responsibility of the clinical study staff at the site under the supervision of the site investigator. The investigator is responsible for ensuring the accuracy, completeness, legibility, and timeliness of the data reported.

All source documents should be completed in a neat, legible manner to ensure accurate interpretation of data.

Hardcopies of the study visit worksheets will be provided for use as source document worksheets for recording data for each participant enrolled in the study. Data recorded in the electronic case report form (eCRF) derived from source documents should be consistent with the data recorded on the source documents.

Clinical data (including AEs), concomitant medications, and expected adverse reactions data) and clinical laboratory data will be entered into iMedNet a 21 CFR Part 11-compliant data capture system provided by the Clinlogix. The data system includes password protection and internal quality checks, such as automatic range checks, to identify data that appear inconsistent, incomplete, or inaccurate. Clinical data will be entered directly from the source documents.

AEs will be coded using MedDRA® dictionary version 12.1 or a more recent version if available. Concomitant medications will be listed by treatment and coded using World Health Organization drug dictionary 2009 Quarter 3 Version or a more recent version if available.

10.1.8.2 STUDY RECORDS RETENTION

Study documents should be retained for a minimum of 2 years after the last approval of a marketing application in an International Conference on Harmonisation (ICH) region and until there are no pending or contemplated marketing applications in an ICH region or until at least 2 years have elapsed since the formal discontinuation of clinical development of the study intervention. These documents should be retained for a longer period, however, if required by local regulations. No records will be destroyed without the written consent of the sponsor, if applicable. It is the responsibility of the sponsor to inform the investigator when these documents no longer need to be retained.

10.1.9 PROTOCOL DEVIATIONS

A protocol deviation is any noncompliance with the clinical study protocol, ICH GCP, or Manual of Procedures (MOP) requirements. The noncompliance may be either on the part of the

participant, the investigator, or the study site staff. As a result of deviations, corrective actions are to be developed by the site and implemented promptly.

These practices are consistent with ICH GCP:

- 4.5 Compliance with Protocol, sections 4.5.1, 4.5.2, and 4.5.3
- 5.1 Quality Assurance and Quality Control, section 5.1.1
- 5.20 Noncompliance, sections 5.20.1, and 5.20.2.

It is the responsibility of the site investigator to use continuous vigilance to identify and report deviations. All deviations must be addressed in study source documents, reported to sponsor. Protocol deviations must be sent to the reviewing IRB per their policies. The site investigator is responsible for knowing and adhering to the reviewing IRB requirements. Further details about the handling of protocol deviations will be included in the MOP.

10.1.10 PUBLICATION AND DATA SHARING POLICY

This study will be registered at ClinicalTrials.gov, and results from this study will be submitted to ClinicalTrials.gov. In addition, every attempt will be made to publish results in peer-reviewed journals.

All proposed publications and presentations by the Investigators or their personnel and associates resulting from or relating to this study must be submitted to Tryp Therapeutics for review and comment before submission for publication or presentation.

Study subjects shall not be identified in any publication or presentation; only study identification numbers shall be used. If photographs are to be used as illustrations, separate informed consent shall be obtained for this purpose, and photographs shall be rendered anonymous.

If the proposed publication or presentation contains patentable subject matter, which, at Tryp Therapeutics discretion, warrants intellectual property protection, Tryp may delay any publication or presentation for up to 60 days after review for the purpose of pursuing such protection. Additional details on the publication policy are listed in the Accelerated Clinical Trial Agreement between Tryp Therapeutics and the University of Florida.

10.1.11 CONFLICT OF INTEREST POLICY

Any actual conflict of interest of persons who have a role in the design, conduct, analysis, publication, or any aspect of this study will be disclosed and managed. Furthermore, persons who have a perceived conflict of interest will be required to have such conflicts managed in a way that is appropriate to their participation in the design and conduct of this study. Tryp Therapeutics has established policies and procedures for all study group members to disclose

all conflicts of interest and will establish a mechanism for the management of all reported dualities of interest.

10.2 ABBREVIATIONS

AAQ-II	Acceptance and Action Questionnaire-II
ACT	Acceptance and Commitment Therapy
ACTH	Adrenocorticotropic Hormone
AE	Adverse Event
BED	Binge Eating Disorder
BES	Binge Eating Scale
BMI	Body Mass Index
BOLD	Blood Oxygen-Level Dependent
BP	Blood Pressure
CBC	Complete Blood Count
C-SSRS	Columbia Suicide Severity Rating Scale
CEQ	Challenging Experiences Questionnaire
CFB	Change from Baseline
CFR	Code of Federal Regulations
CGI-I	Clinical Global Impression-Improvement Scale
CMP	Clinical Monitoring Plan
CRF	Case Report Form
CRO	Clinical Research Organization
DEA	Drug Enforcement Agency
DSM-5	Diagnostic and Statistical Manual of Mental Disorders 5th Edition
EBI	Emotional Breakthrough Inventory
EC	Ethics Committee
ECG	Electrocardiogram
eCRF	Electronic Case Report Forms
EEG	Electroencephalogram
FAS	Full Analysis Set
FDA	Food and Drug Administration
fMRI	Functional Magnetic Resonance Imaging
GCP	Good Clinical Practice
GLP	Good Laboratory Practices
HADS	Hospital Anxiety and Depression Scale
HOMA-IR	Homeostatic Model Assessment of Insulin Resistance
HPMC	Hydroxypropyl Methyl Cellulose
HPPD	Hallucinogen Persisting Perception Disorder
HR	Heart Rate

IB	Investigator's Brochure
ICH	International Conference on Harmonisation
ICH GCP	International Conference on Harmonisation Good Clinical Practice
IEC	Independent Ethics Committee
IND	Investigational New Drug
IRB	Institutional Review Board
MAOI	Monoamine Oxidase Inhibitor
MedDRA	Medical Dictionary for Regulatory Activities
MEQ30	Mystical Experience Questionnaire (30 questions)
MOP	Manual of Procedures
MRS	Monitor Rating Scale
NCT	National Clinical Trial
OCD	Obsessive Compulsive Disorder
PGI-I	Patient Global Impression Improvement
PI	Principal Investigator
PP	Per Protocol
PRN	As needed
QC	Quality Control
QTc	Corrected QT Interval
QTcF	Fridericia's Correction Formula of the QT Interval
SAE	Serious Adverse Event
SAP	Statistical Analysis Plan
SD	Standard Deviation
SOA	Schedule of Activities
SOP	Standard Operating Procedure
SRC	Safety Review Committee
SSRI	Selective Serotonin Reuptake Inhibitor
TEAE	Treatment-Emergent Adverse Event
TRP-8802	psilocybin drug substance prepared according to current manufacturing processes at Usona Institute which supplies the psilocybin to Tryp Therapeutics Inc
US	United States
VAS	Visual analog scale

APPENDIX A: DOSING AND THERAPY SESSIONS

The following is a summary of the Set and Setting sessions that will occur at pre-dosing, dosing, and post dosing. All staff will be trained to the separately-provided Psychotherapy Training Manual.

Overview of Therapy Sessions

1. Preparatory Session 1 (1 week prior to medication session)	In-person or video<br><br>Lead therapist	Therapist aims to establish therapeutic alliance through:<br>• Building rapport and therapeutic alliance<br>• Listening to participant's narrative of binge eating, anxiety, guilt, etc., and treatment history to understand patterns of psychological inflexibility that are most prominent<br>• Psychoeducation regarding the TRP-8802 experience and Acceptance and Commitment Therapy (ACT)
2. Preparatory Session 2 (1-3 days before medication session)	In-person or video<br><br>Both therapists	• Teaching grounding techniques including abdominal breathing and dropping anchor<br>• Discussion of therapeutic boundaries (e.g., touch) and safety measures<br>• Assisting the participant in setting a motivation for medication session
3. Medication session (≥8 hrs)	In-person<br><br>Both therapists	• In line with supportive stance during the medication session, the therapist does not provide significant ACT interventions or feedback<br>• ACT-based clinical formulation continues as therapist listens to emergent narratives and notes instances of psychological flexibility and inflexibility, especially present moment awareness, avoidance, and values
4. Integration session 1 (day after medication session)	In-person<br><br>Both therapists	• Therapist elicits complete narrative of participant's experience during medication session<br>• Focused ACT-based clinical activity related to contacting the present moment, defusion, or acceptance based on clinical formulation (time permitting).
5. Integration session 2 (~7 days after medication session)	Video or In-person<br><br>Both or one therapist	Therapist and participant continue to review and reflect on the participant's:<br>• TRP-8802 experience, including any emotional, mental, or lifestyle changes that followed the dosing session<br>• Focused ACT-based clinical activity related to contacting the present moment, defusion, or acceptance based on clinical formulation.
6. Therapy follow-up session (~2 weeks after medication session)	Video or In-person<br><br>Lead therapist	Therapist continues to explore and reinforce:<br>• Insights gained from the TRP-8802 experience while assessing for changes in psychological flexibility<br>• How the dosing and therapy sessions brought each ACT process to light<br>• Successful behavioral changes and committed actions taken<br>• Mindfulness practices and other concrete ways the study experience can be translated into lasting changes<br>• Therapist leads termination discussion and plans for follow-up care for the participant.

11 REFERENCES

Bailer, U. F., & Kaye, W. H. (2010). Serotonin: Imaging Findings in Eating Disorders. In (pp. 59-79) Behavioral Neurobioloby of Eating Disorders. Springer Berlin Heidelberg. *https://doi.org/10.1007/7854_2010_78*

Barrett, F. S., Bradstreet, M. P., Leoutsakos, J.-M. S., Johnson, M. W., & Griffiths, R. R. (2016). The Challenging Experience Questionnaire: Characterization of challenging experiences with psilocybin mushrooms. *Journal of Psychopharmacology*, *30*(12), 1279-1295. *https://doi.org/10.1177/0269881116678781*

Barrett, F. S., Johnson, M. W., & Griffiths, R. R. (2015). Validation of the revised Mystical Experience Questionnaire in experimental sessions with psilocybin. *Journal of Psychopharmacology*, *29*(11), 1182-1190. *https://doi.org/10.1177/0269881115609019*

Bond, F. W., Hayes, S. C., Baer, R. A., Carpenter, K. M., Guenole, N., Orcutt, H. K., Waltz, T., & Zettle, R. D. (2011). Preliminary Psychometric Properties of the Acceptance and Action Questionnaire-II: a Revised Measure of Psychological Inflexibility and Experiential Avoidance. *Behav Ther*, *42*(4), 676-688. *https://doi.org/10.1016/j.beth.2011.03.007*

Boswell, R. G., Potenza, M. N., Grilo, C. M. (2021). The Neurobiology of Binge-eating Disorder Compared with Obesity: Implications for Differential Therapeutics. *Clinical Therapeutics,* 43. https://doi.org/10.1016/j.clinthera.2020.10.014

Brown, R. T., Nicholas, C. R., Cozzi, N. V., Gassman, M. C., Cooper, K. M., Muller, D., Thomas, C. D., Hetzel, S. J., Henriquez, K. M., Ribaudo, A. S., & Hutson, P. R. (2017). Pharmacokinetics of Escalating Doses of Oral Psilocybin in Healthy Adults. *Clin Pharmacokinet*, *56*(12), 1543-1554. *https://doi.org/10.1007/s40262-017-0540-6*

Carhart-Harris, R. L., Leech, R., Hellyer, P. J., Shanahan, M., Feilding, A., Tagliazucchi, E., Chialvo, D. R., & Nutt, D. (2014). The entropic brain: a theory of conscious states informed by neuroimaging research with psychedelic drugs. *Frontiers in Human Neuroscience*, *8*. *https://doi.org/10.3389/fnhum.2014.00020*

Carhart-Harris, R. L., & Nutt, D. J. (2013). Experienced drug users assess the relative harms and benefits of drugs: a web-based survey. *J Psychoactive Drugs*, *45*(4), 322-328. *https://doi.org/10.1080/02791072.2013.825034*

Carhart-Harris, R., Giribaldi, B., Watts, R., Nutt, D.J. (2021) Trial of Psilocybin versus Escitalopram for Depression. *N Engl J Med.*, 384:1402-11.

Chen, J., Li, M., Yan, X., Wu, E., Zhu, H., Lee, K. J., Chu, V. M., Zhan, L., Lee, W., & Kang, J. S. (2011). Determining the pharmacokinetics of psilocin in rat plasma using ultra-performance liquid chromatography coupled with a photodiode array detector after orally administering an extract of Gymnopilus spectabilis. *J Chromatogr B Analyt Technol Biomed Life Sci*, *879*(25), 2669-2672. *https://doi.org/10.1016/j.jchromb.2011.07.003*

Citrome, L. (2019). Binge eating disorder revisited: what's new, what's different, what's next. *CNS Spectr*, *24*(S1), 4-13. *https://doi.org/10.1017/s1092852919001032*

Dahmane, E., Hutson, P. R., & Gobburu, J. V. S. (2021). Exposure-Response Analysis to Assess the Concentration-QTc Relationship of Psilocybin/Psilocin. *Clin Pharmacol Drug Dev*, *10*(1), 78-85. *https://doi.org/10.1002/cpdd.796*

Davis, A. K., Barrett, F. S., May, D. G., Cosimano, M. P., Sepeda, N. D., Johnson, M. W., Finan, P. H., & Griffiths, R. R. (2021). Effects of Psilocybin-Assisted Therapy on Major Depressive Disorder. *JAMA Psychiatry*, *78*(5), 481. *https://doi.org/10.1001/jamapsychiatry.2020.3285*

Donnelly, B., Touyz, S., Hay, P., Burton, A., Russell, J., Caterson, I. (2018). Neuroimaging in bulimia nervosa and binge eating disorder: a systematic review. *J. Eating Disorders*, 6:3. DOI/10.1186/s40337-018-0187-1

Erritzoe, D., Frokjaer, V. G., Haugbol, S., Marner, L., Svarer, C., Holst, K., Baaré, W. F. C., Rasmussen, P. M., Madsen, J., Paulson, O. B., Knudsen, G. M. (2009) Brain serotonin 2A receptor binding: Relations to body mass index, tobacco and alcohol use. *Neuroimage*, 46(1):23-30. doi: 10.1016/j.neuroimage.2009.01.050.

Espiard, M. L., Lecardeur, L., Abadie, P., Halbecq, I., & Dollfus, S. (2005). Hallucinogen persisting perception disorder after psilocybin consumption: a case study. *Eur Psychiatry*, *20*(5-6), 458-460. *https://doi.org/10.1016/j.eurpsy.2005.04.008*

Fairburn, C. G., & Beglin, S. J. (1994). Assessment of eating disorders: interview or self-report questionnaire? *Int J Eat Disord*, *16*(4), 363-370.

Foldi, C. J., Liknaitzky, P., Williams, M., & Oldfield, B. J. (2020). Rethinking Therapeutic Strategies for Anorexia Nervosa: Insights From Psychedelic Medicine and Animal Models. *Frontiers in Neuroscience*, *14*. https://doi.org/10.3389/fnins.2020.00043

Geyer, M. A., & Vollenweider, F. X. (2008). Serotonin research: contributions to understanding psychoses. *Trends Pharmacol Sci*, *29*(9), 445-453. https://doi.org/10.1016/j.tips.2008.06.006

Gormally, J., Black, S., Daston, S., & Rardin, D. (1982). The assessment of binge eating severity among obese persons. *Addict Behav*, *7*(1), 47-55. https://doi.org/10.1016/0306-4603(82)90024-7

Gouzoulis-Mayfrank, E., Thelen, B., Habermeyer, E., Kunert, H. J., Kovar, K. A., Lindenblatt, H., Hermle, L., Spitzer, M., & Sass, H. (1999). Psychopathological, neuroendocrine and autonomic effects of 3,4-methylenedioxyethylamphetamine (MDE), psilocybin and d-methamphetamine in healthy volunteers. Results of an experimental double-blind placebo-controlled study. *Psychopharmacology (Berl)*, *142*(1), 41-50. https://doi.org/10.1007/s002130050860

Griffiths, R. R., Johnson, M. W., Carducci, M. A., Umbricht, A., Richards, W. A., Richards, B. D., Cosimano, M. P., & Klinedinst, M. A. (2016). Psilocybin produces substantial and sustained decreases in depression and anxiety in patients with life-threatening cancer: A randomized double-blind trial. *Journal of Psychopharmacology*, *30*(12), 1181-1197. https://doi.org/10.1177/0269881116675513

Griffiths, R. R., Johnson, M. W., Richards, W. A., Richards, B. D., McCann, U., & Jesse, R. (2011). Psilocybin occasioned mystical-type experiences: immediate and persisting dose-related effects. *Psychopharmacology*, *218*(4), 649-665. https://doi.org/10.1007/s00213-011-2358-5

Griffiths, R. R., Richards, W. A., McCann, U., & Jesse, R. (2006). Psilocybin can occasion mystical-type experiences having substantial and sustained personal meaning and spiritual significance. *Psychopharmacology (Berl)*, *187*(3), 268-283; discussion 284-292. https://doi.org/10.1007/s00213-006-0457-5

Grignaschi, G., Sironi, F., & Samanin, R. (1996). Stimulation of 5-HT2A receptors in the paraventricular hypothalamus attenuates neuropeptide Y-induced hyperphagia through activation of corticotropin releasing factor. *Brain Res*, *708*(1-2), 173-176. https://doi.org/10.1016/0006-8993(95)01373-3

Guerdjikova, A. Romo-Nava, Francisco, Blom, T.J., Mori, N., McElroy, S.L. (2021) Study protocol and rationale for a randomized, placebo-controlled trial of solriamfetol to treat bing eating disorder. *Contemporary Clinical Trials* , *110,* 106587. DOI: 10.1016/j.cct.2021.106587

Guerdjikova, A. I., Mori, N., Casuto, L. S., & McElroy, S. L. (2019). Update on Binge Eating Disorder. *Med Clin North Am*, *103*(4), 669-680. https://doi.org/10.1016/j.mcna.2019.02.003

Guy, W. (1976). ECDEU assessment manual for psychopharmacology U.S. Dept. of Health, Education, and Welfare, Public Health Service, Alcohol, Drug Abuse, and Mental Health Administration, National Institute of Mental Health, Psychopharmacology Research Branch, Division of Extramural Research Programs.

Hasler, F., Bourquin, D., Brenneisen, R., Bär, T., & Vollenweider, F. X. (1997). Determination of psilocin and 4-hydroxyindole-3-acetic acid in plasma by HPLC-ECD and pharmacokinetic profiles of oral and intravenous psilocybin in man. *Pharm Acta Helv*, *72*(3), 175-184. https://doi.org/10.1016/s0031-6865(97)00014-9

Hasler, F., Grimberg, U., Benz, M. A., Huber, T., & Vollenweider, F. X. (2004). Acute psychological and physiological effects of psilocybin in healthy humans: a double-blind, placebo-controlled dose-effect study. *Psychopharmacology (Berl)*, *172*(2), 145-156. https://doi.org/10.1007/s00213-003-1640-6

Hopf, A., & Eckert, H. (1974). Distribution patterns of 14-C-psilocin in the brains of various animals. *Act Nerv Super (Praha)*, *16*(1), 64-66.

Horita, A., & Weber, L. J. (1961). The enzymic dephosphorylation and oxidation of psilocybin and psilocin by mammalian tissue homogenates. *Biochem Pharmacol*, *7*, 47-54. https://doi.org/10.1016/0006-2952(61)90124-1

Houazene, S., Leclerc, J. B., O'Connor, K., & Aardema, F. (2021). "Shame on you": The impact of shame in body-focused repetitive behaviors and binge eating. *Behav Res Ther*, *138*, 103804. https://doi.org/10.1016/j.brat.2021.103804

Johnson, M., Richards, W., & Griffiths, R. (2008). Human hallucinogen research: guidelines for safety. *Journal of Psychopharmacology*, *22*(6), 603-620. https://doi.org/10.1177/0269881108093587

Johnson MW, Griffiths RR. (2017) *Potential Therapeutic Effects of Psilocybin.* Neurotherapeutics, 14(3):734-740. DOI: 10.1007/s13311-017-0542-y

Kalberer, F., Kreis, W., & Rutschmann, J. (1962). The fate of psilocin in the rat. *Biochem Pharmacol*, *11*, 261-269. https://doi.org/10.1016/0006-2952(62)90050-3

Lafrance, A., Loizaga-Velder, A., Fletcher, J., Renelli, M., Files, N., & Tupper, K. W. (2017). Nourishing the Spirit: Exploratory Research on Ayahuasca Experiences along the Continuum of Recovery from Eating Disorders. *J Psychoactive Drugs*, *49*(5), 427-435. https://doi.org/10.1080/02791072.2017.1361559

Lutter, M., & Nestler, E. J. (2009). Homeostatic and Hedonic Signals Interact in the Regulation of Food Intake. *J Nutr*, *139*(3), 629-632. https://doi.org/10.3945/jn.108.097618

Lyons, T., & Carhart-Harris, R. L. (2018). More Realistic Forecasting of Future Life Events After Psilocybin for Treatment-Resistant Depression. *Frontiers in Psychology*, *9*. https://doi.org/10.3389/fpsyg.2018.01721

Maclean, K. A., Johnson, M. W., & Griffiths, R. R. (2011). Mystical experiences occasioned by the hallucinogen psilocybin lead to increases in the personality domain of openness. *Journal of Psychopharmacology*, *25*(11), 1453-1461. https://doi.org/10.1177/0269881111420188

Madsen, M. K., Fisher, P. M., Burmester, D., Dyssegaard, A., Stenbæk, D. S., Kristiansen, S., Johansen, S. S., Lehel, S., Linnet, K., Svarer, C., Erritzoe, D., Ozenne, B., & Knudsen, G. M. (2019). Psychedelic effects of psilocybin correlate with serotonin 2A receptor occupancy and plasma psilocin levels. *Neuropsychopharmacology*, *44*(7), 1328-1334. https://doi.org/10.1038/s41386-019-0324-9

McElroy, S. L., Hudson, J. I., Mitchell, J., E., Wilfley, D., Ferreira-Cornwell, M. C., Gao, J., Wang, J., Whitaker, T., Jonas, J., Gasior, M. (2015) Efficacy and Safety of Lisdexamfetamine for Treatment of Adults with Moderate to Severe Binge-Eating Disorder. A Randomized Clinical Trial. *JAMA Psychiatry,* 72, 236. DOI: 10.1038/npp.2015.275

Metzner, R. (2005). *Sacred Mushroom of Vision: Teonanacatl*. Park Street Press.

Nicholas, C. R., Henriquez, K. M., Gassman, M. C., Cooper, K. M., Muller, D., Hetzel, S., Brown, R. T., Cozzi, N. V., Thomas, C., Hutson, P. R. (2018). High dose psilocybin is associated with positive subjective effects in healthy volunteers. *Journal of Phychopharmacology*, 32 (7), 770-778. https://doi:10.1177/0269881118780713.

Nichols, D. E. (2004). Hallucinogens. *Pharmacol Ther*, *101*(2), 131-181. https://doi.org/10.1016/j.pharmthera.2003.11.002

Nutt, D. J., King, L. A., & Phillips, L. D. (2010). Drug harms in the UK: a multicriteria decision analysis. *Lancet*, *376*(9752), 1558-1565. https://doi.org/10.1016/s0140-6736(10)61462-6

Odland AU, Kristensen JL, Andreasen JT (2021) Investigating the role of 5-HT2A and 5-HT2C receptor activation in the effects of psilocybin, DOI, and citalopram on marble burying in mice. Behav Brain Res. ;401:113093. DOI: 10.1016/j.bbr.2020.113093

Passie, T., Seifert, J., Schneider, U., & Emrich, H. M. (2002). The pharmacology of psilocybin. *Addict Biol*, *7*(4), 357-364. https://doi.org/10.1080/1355621021000005937

Posner, K., Brown, G. K., Stanley, B., Brent, D. A., Yershova, K. V., Oquendo, M. A., Currier, G. W., Melvin, G. A., Greenhill, L., Shen, S., & Mann, J. J. (2011). The Columbia–Suicide Severity Rating Scale: Initial Validity and Internal Consistency Findings From Three Multisite Studies With Adolescents and Adults. *American Journal of Psychiatry*, *168*(12), 1266-1277. https://doi.org/10.1176/appi.ajp.2011.10111704

Renelli, M., Fletcher, J., Tupper, K. W., Files, N., Loizaga-Velder, A., & Lafrance, A. (2020). An exploratory study of experiences with conventional eating disorder treatment and ceremonial ayahuasca for the healing of eating disorders. *Eat Weight Disord*, *25*(2), 437-444. https://doi.org/10.1007/s40519-018-0619-6

Romeo B, Hermand M, Pétillion A, Karila L, Benyamina A.J (2021) Clinical and biological predictors of psychedelic response in the treatment of psychiatric and addictive disorders: A systematic review. Psychiatr Res. 137:273-282. https://doi.org/10.1016/j.jpsychires.2021.03.002

Roseman, L., Haijen, E., Idialu-Ikato, K., Kaelen, M., Watts, R., & Carhart-Harris, R. (2019). Emotional breakthrough and psychedelics: Validation of the Emotional Breakthrough Inventory. *J Psychopharmacol*, *33*(9), 1076-1087. https://doi.org/10.1177/0269881119855974

Ross, S., Bossis, A., Guss, J., Agin-Liebes, G., Malone, T., Cohen, B., Mennenga, S. E., Belser, A., Kalliontzi, K., Babb, J., Su, Z., Corby, P., & Schmidt, B. L. (2016). Rapid and sustained symptom reduction following psilocybin treatment for anxiety and depression in patients with life-threatening cancer: a randomized controlled trial. *Journal of Psychopharmacology*, *30*(12), 1165-1180. https://doi.org/10.1177/0269881116675512

Rucker, J. J. H., Iliff, J., & Nutt, D. J. (2018). Psychiatry & the psychedelic drugs. Past, present & future. *Neuropharmacology*, *142*, 200-218. https://doi.org/10.1016/j.neuropharm.2017.12.040

Samodien, E., & Chellan, N. (2021). Hypothalamic neurogenesis and its implications for obesity-induced anxiety disorders. *Front Neuroendocrinol*, *60*, 100871. https://doi.org/10.1016/j.yfrne.2020.100871

Serretti, A., Drago, A., & De Ronchi, D. (2007). HTR2A Gene Variants and Psychiatric Disorders: a Review of current Literature and Selection of SNPs for Future Studies. *Curr Med Chem*, *14*(19), 2053-2069. https://doi.org/10.2174/092986707781368450

Spriggs, M. J., Kettner, H., & Carhart-Harris, R. L. (2021). Positive effects of psychedelics on depression and wellbeing scores in individuals reporting an eating disorder. *Eat Weight Disord*, *26*(4), 1265-1270. https://doi.org/10.1007/s40519-020-01000-8

Studerus, E., Kometer, M., Hasler, F., & Vollenweider, F. X. (2011). Acute, subacute and long-term subjective effects of psilocybin in healthy humans: a pooled analysis of experimental studies. *J Psychopharmacol*, *25*(11), 1434-1452. https://doi.org/10.1177/0269881110382466

Sugimoto, Y., Yoshikawa, T., Noma, T., & Yamada, J. (2001). The 5-HT2C/2B Receptor Agonist m-Chlorophenylpiperazine (mCPP) Inhibits 2-Deoxy-D-glucose (2-DG)-Induced Hyperphagia in Rats. *Biological and Pharmaceutical Bulletin*, *24*(12), 1431-1433. https://doi.org/10.1248/bpb.24.1431

Tryp Therapeutics. (2021, v2.1). Psilocybin, [3-[2-(dimethylamino) ethyl]-1H-indol-4-yl] dihydrogen phosphate Investigator's Brochure.

Tylš, F., Páleníček, T., & Horáček, J. (2014). Psilocybin--summary of knowledge and new perspectives. *Eur Neuropsychopharmacol*, *24*(3), 342-356. https://doi.org/10.1016/j.euroneuro.2013.12.006

Zigmond, A. S., & Snaith, R. P. (1983). The Hospital Anxiety and Depression Scale. *Acta Psychiatr Scand*, *67*(6), 361-370. https://doi.org/10.1111/j.1600-0447.1983.tb09716.x
