# Supplementary material for: An open-label pilot study of psilocybin-assisted therapy for binge eating disorder
Source: J Eat Disord. 2026 Jan 3;14:41. doi: 10.1186/s40337-025-01508-3 (PMC12857019; doi:10.1186/s40337-025-01508-3)
Supplement: Supplementary file 2 — Supplementary Material 2 [file 40337_2025_1508_MOESM2_ESM.docx]

**Supplement 2.**

*Biomarker results at each timepoint*

| Participant | Week | Biomarker | Result |
| --- | --- | --- | --- |
| P1 | 2 | Leptin | 47.3 |
| P1 | 2 | Adiponectin | 12 |
| P1 | 2 | Ghrelin | 556 |
| P1 | 2 | Insulin | 28.65 |
| P1 | 2 | Glucose fasting | 89 |
| P1 | 2 | HOMA-IR | 6.3 |
| P1 | 6 | Leptin | 49.9 |
| P1 | 6 | Adiponectin | 13 |
| P1 | 6 | Ghrelin | 213 |
| P1 | 6 | Insulin | 41.06 |
| P1 | 6 | Glucose fasting | 85 |
| P1 | 6 | HOMA-IR | 8.6 |
| P1 | 10 | Leptin | 39.2 |
| P1 | 10 | Adiponectin | 9 |
| P1 | 10 | Ghrelin | 695 |
| P1 | 10 | Insulin | 112.95 |
| P1 | 10 | Glucose fasting | 105 |
| P1 | 10 | HOMA-IR | 29.3 |
| P1 | 14 | Leptin | 54.8 |
| P1 | 14 | Adiponectin | 11 |
| P1 | 14 | Ghrelin | 753 |
| P1 | 14 | Insulin | 40.94 |
| P1 | 14 | Glucose fasting | 82 |
| P1 | 14 | HOMA-IR | 8.3 |
| P2 | 2 | Leptin | 18.8 |
| P2 | 2 | Adiponectin | 4 |
| P2 | 2 | Ghrelin | 249 |
| P2 | 2 | Insulin | 79.26 |
| P2 | 2 | Glucose fasting | 266 |
| P2 | 2 | HOMA-IR | 52.1 |
| P2 | 6 | Leptin | 16.3 |
| P2 | 6 | Adiponectin | 3 |
| P2 | 6 | Ghrelin |  |
| P2 | 6 | Insulin | 85.94 |
| P2 | 6 | Glucose fasting | 189 |
| P2 | 6 | HOMA-IR | 40.1 |
| P2 | 10 | Leptin | 15.8 |
| P2 | 10 | Adiponectin | 3 |
| P2 | 10 | Ghrelin | 456 |
| P2 | 10 | Insulin | 46.42 |
| P2 | 10 | Glucose fasting | 286 |
| P2 | 10 | HOMA-IR | 32.8 |
| P2 | 14 | Leptin | 14.6 |
| P2 | 14 | Adiponectin | 4 |
| P2 | 14 | Ghrelin | 244 |
| P2 | 14 | Insulin | 66.51 |
| P2 | 14 | Glucose fasting | 213 |
| P2 | 14 | HOMA-IR | 35 |
| P3 | 2 | Leptin | 1.8 |
| P3 | 2 | Adiponectin | 45 |
| P3 | 2 | Ghrelin | 3145 |
| P3 | 2 | Insulin | 2.87 |
| P3 | 2 | Glucose fasting | 93 |
| P3 | 2 | HOMA-IR | 0.7 |
| P3 | 6 | Leptin | 2 |
| P3 | 6 | Adiponectin | 38 |
| P3 | 6 | Ghrelin | 1279 |
| P3 | 6 | Insulin | 3.73 |
| P3 | 6 | Glucose fasting | 91 |
| P3 | 6 | HOMA-IR | 0.8 |
| P3 | 10 | Leptin | 1 |
| P3 | 10 | Adiponectin | 41 |
| P3 | 10 | Ghrelin | 1748 |
| P3 | 10 | Insulin | 1.78 |
| P3 | 10 | Glucose fasting | 84 |
| P3 | 10 | HOMA-IR | 0.4 |
| P3 | 14 | Leptin | 2.9 |
| P3 | 14 | Adiponectin | 38 |
| P3 | 14 | Ghrelin |  |
| P3 | 14 | Insulin | 2.52 |
| P3 | 14 | Glucose fasting | 85 |
| P3 | 14 | HOMA-IR | 0.5 |
| P5 | 2 | Leptin | 21.2 |
| P5 | 2 | Adiponectin | 14 |
| P5 | 2 | Ghrelin | 1244 |
| P5 | 2 | Insulin | 7.5 |
| P5 | 2 | Glucose fasting | 94 |
| P5 | 2 | HOMA-IR | 1.7 |
| P5 | 6 | Leptin | 21.2 |
| P5 | 6 | Adiponectin | 17 |
| P5 | 6 | Ghrelin | 1025 |
| P5 | 6 | Insulin | 5.64 |
| P5 | 6 | Glucose fasting | 109 |
| P5 | 6 | HOMA-IR | 1.5 |
| P5 | 10 | Leptin | 39.7 |
| P5 | 10 | Adiponectin | 9.3 |
| P5 | 10 | Ghrelin | 989 |
| P5 | 10 | Insulin | 6.55 |
| P5 | 10 | Glucose fasting | 105 |
| P5 | 10 | HOMA-IR | 1.7 |
| P5 | 14 | Leptin | 32 |
| P5 | 14 | Adiponectin | 17 |
| P5 | 14 | Ghrelin | 385 |
| P5 | 14 | Insulin | 12.23 |
| P5 | 14 | Glucose fasting | 113 |
| P5 | 14 | HOMA-IR | 3.4 |
| P6 | 2 | Leptin | 57.5 |
| P6 | 2 | Adiponectin | 7 |
| P6 | 2 | Ghrelin | 771 |
| P6 | 2 | Insulin | 18.32 |
| P6 | 2 | Glucose fasting | 88 |
| P6 | 2 | HOMA-IR | 4 |
| P6 | 6 | Leptin | 52.4 |
| P6 | 6 | Adiponectin | 6 |
| P6 | 6 | Ghrelin | 815 |
| P6 | 6 | Insulin | 20.02 |
| P6 | 6 | Glucose fasting | 82 |
| P6 | 6 | HOMA-IR | 4.1 |
| P6 | 10 | Leptin | 55.3 |
| P6 | 10 | Adiponectin | 5 |
| P6 | 10 | Ghrelin |  |
| P6 | 10 | Insulin | 14.22 |
| P6 | 10 | Glucose fasting | 89 |
| P6 | 10 | HOMA-IR | 3.1 |
| P6 | 14 | Leptin | 45.4 |
| P6 | 14 | Adiponectin | 3.7 |
| P6 | 14 | Ghrelin | 647 |
| P6 | 14 | Insulin | 24.67 |
| P6 | 14 | Glucose fasting | 79 |
| P6 | 14 | HOMA-IR | 3.1 |

Note: Leptin: ng/mL; Adiponectin: ug/mL, Ghrelin: pg/mL; Insulin Total – Plasma Fasting: mciu/ml; Glucose fasting: mg/dl; HOMA-IR, Homeostatic Model Assessment of Insulin Resistance: no unit, ratio
